# Therapeutic application of PPE2 protein of *Mycobacterium tuberculosis* in inhibiting tissue inflammation

**DOI:** 10.15252/emmm.202114891

**Published:** 2022-07-11

**Authors:** Ravi Pal, Madhu Babu Battu, Sangita Mukhopadhyay

**Affiliations:** ^1^ Laboratory of Molecular Cell Biology Center for DNA Fingerprinting and Diagnostics (CDFD) Hyderabad Telangana India; ^2^ Graduate Studies Manipal Academy of Higher Education Manipal Karnataka India

**Keywords:** fibroblast, inflammation/tissue injury, mast cell, PPE2 protein/peptide, SCF, Immunology, Pharmacology & Drug Discovery

## Abstract

There is an increasing need to develop biological anti‐inflammatory agents that are more targeted, effective, and with lesser side effects as compared to conventional chemical drugs. In the present study, we found that *Mycobacterium tuberculosis* protein PPE2 and a synthetic derivative peptide can suppress the mast cell population and inhibit several vasoactive and fibrogenic mediators and pro‐inflammatory cytokines induced by mast cells in formalin‐induced tissue injury. PPE2 was found to inhibit transcription from the promoter of stem cell factor, important for mast cell maintenance and migration. Thus, PPE2/peptide can be used as a potent nonsteroidal therapeutic agent for the treatment of inflammation and tissue injury.

## Graphical abstract

Mast cells are critical for sensing tissue damage and triggering inflammatory response around the injury. Tissue fibroblasts secrete stem cell factor (SCF) and regulate mast cell population. PPE2 binds to the SCF promoter and inhibits SCF transcription, leading to reduced mast cell population and subside inflammation. A PPE2‐derived peptide also demonstrated anti‐inflammatory properties similar to the whole protein.

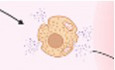

## Introduction

Inflammation is a generic protective biological response toward a harmful stimulus (pathogens, damaged cells, or irritants). Tissue inflammation results in vasodilation followed by the immigration of leukocytes and plasma, resulting in redness, swelling, and pain. Inflammation helps to eliminate the initial causes and clears out necrotic cells and tissues. Local inflammatory responses are beneficial, but at times, they might be harmful to the body. Chronic inflammation leads to greater immigration of immune cells followed by the destruction of tissues, resulting in a pathological condition. Some classical examples of chronic inflammation are rheumatoid arthritis, periodontitis, atherosclerosis, and even cancer (e.g., gallbladder carcinoma).

In most of cases, steroidal and nonsteroidal anti‐inflammatory drugs (NSAIDs) are used for treating inflammation. Steroids, mainly glucocorticoids, represent the standard therapy for inflammatory diseases such as asthma, rheumatoid arthritis, inflammatory bowel disease, and autoimmune disorders. Glucocorticoids bind to glucocorticoid receptors and upregulate transcription of anti‐inflammatory genes, for example, *il‐10* and *il‐1β* (Barnes, 1998). NSAIDs, like Aspirin, and Diclofenac suppress inflammation by inhibiting cyclooxygenase activity (Inoue *et al*, 2013). Both steroidal and nonsteroidal anti‐inflammatory drugs are quite effective but are associated with adverse side effects, especially when used for longer (Marcum & Hanlon, 2010; Wong, 2019).

Irrespective of the cause of inflammation (physical, chemical, or biological), tissue‐resident cells are the first ones to sense the abnormality in the microenvironment and signals for generating appropriate responses. Neighboring mast cells are the first cells to sense injury and necrotic tissue (Lunderius‐Andersson *et al*, 2012). The role of mast cells in inflammation has been studied extensively (Krystel‐Whittemore *et al*, 2015). Necrotic cells activate mast cells by secreting IL‐33 (Enoksson *et al*, 2011). Once activated, mast cells release/degranulate anaphylactic mediators/compounds into the local microenvironment (Theoharides *et al*, 2012), which promotes extravasation of leukocytes and plasma, causing redness, swelling, and pain. Mast cell‐deficient mice fail to develop arthritis (Nigrovic & Lee, 2005), indicating its important role in inflammation. Therefore, reduction of mast cell activity or mast cell population could be an effective strategy to treat inflammation and its related disorders.

In this study, we show that the administration of recombinantly purified PPE2 protein (rPPE2) or the synthetic peptide derived from PPE2 was able to reduce formalin‐induced paw inflammation in Balb/c mice. We observed that rPPE2/peptide administration depletes the mast cell population and thereby reduces mast cell‐specific mediators in inflamed paw tissues. We showed that PPE2 inhibits the transcription of the stem cell factor (SCF), which is indispensable for *in situ* survival of tissue‐resident mast cells (Finotto *et al*, 1997) as well as for their immigration from the peripheral blood into the tissues (Huang *et al*, 2008). To the best of our knowledge, we, for the first time, have identified a protein and/or peptide that suppress inflammation by inhibiting the mast cell in the paw tissue. As the biologics and immune selective anti‐inflammatory derivatives are the promising drug classes that will play the main role in the market, the present study may be important in identifying a novel molecule with a potent effect in suppressing inflammatory symptoms and tissue injury.

## Results

### rPPE2 subsides paw inflammation in mice

**Figure 1:**
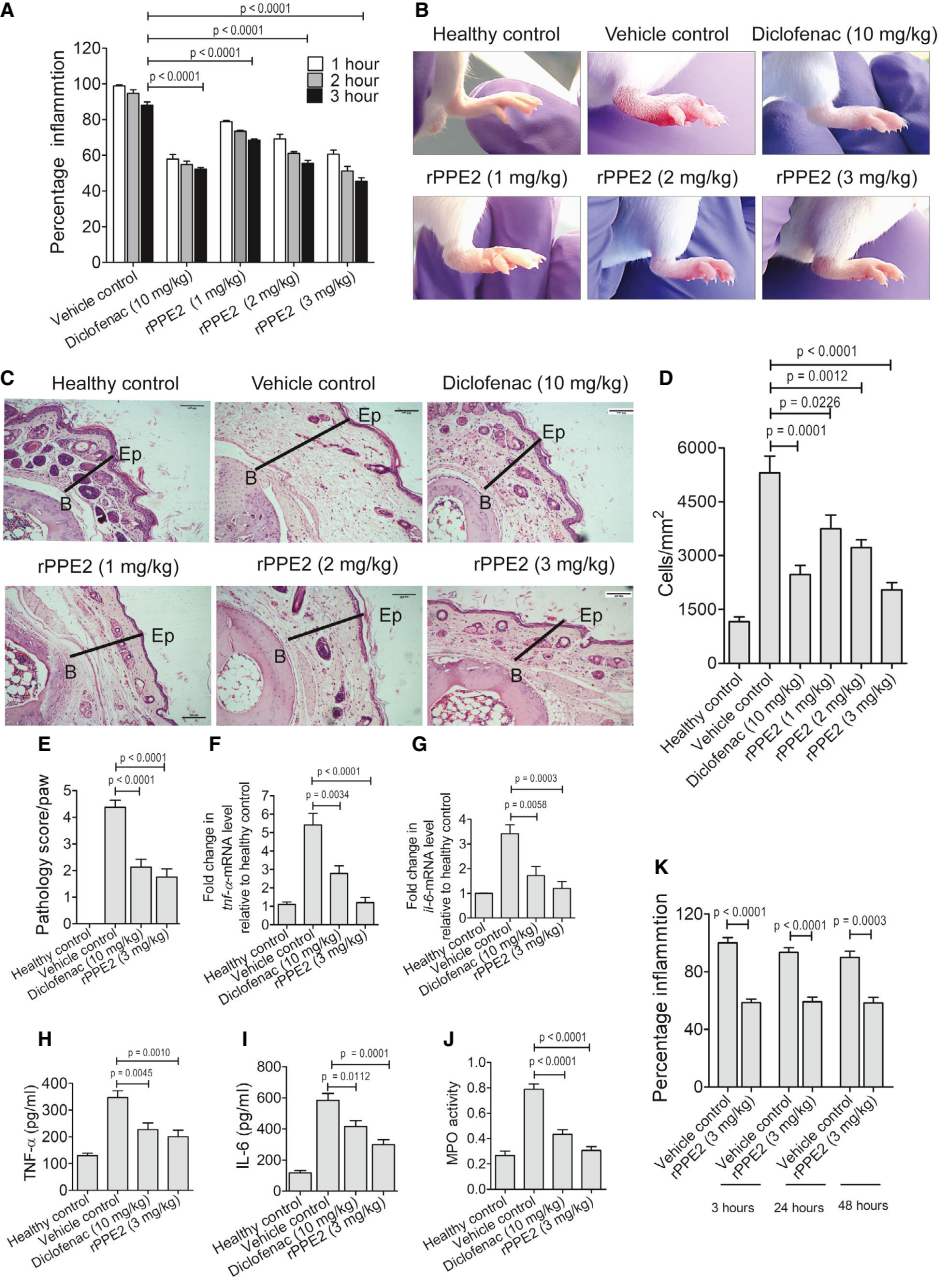
PPE2 subsides paw inflammation in mice A–JA subplantar injection of 5% of formalin (20 μl) was given in the right hind paw of Balb/c mice, and an equal volume of PBS was injected in the left hind paw. After 1 h of development of edema, mice were administered intraperitoneally with a single dose of either PBS (vehicle control) or Diclofenac (10 mg/kg) or different concentrations of rPPE2 (1 or 2 or 3 mg/kg). (A) Graphical representation of percentage inflammation in paw (paw thickness) was shown. Data were analyzed using one‐way ANOVA with Bonferroni *post hoc* test. (B) Representative photographs of inflamed paws after 3 h of treatment. (C) Also, after 3‐h post‐treatment, mice were sacrificed; the paw sections were prepared and stained with hematoxylin and eosin. Photographs of representative sections were visualized at 20× magnification (scale bar = 100 μm). The solid line represents thickness/edema (B, bone; Ep, epidermis). (D) The cells were counted using ImageJ software and plotted as cells per mm^2^ (cells/mm^2^). (E) Formalin (20 μl) was injected in the hind paw of Balb/c mice. After 1 h of development of edema, mice were administered intraperitoneally with a single dose of either PBS (vehicle control) or Diclofenac (10 mg/kg) or rPPE2 (3 mg/kg) and pathology score was calculated after 3 h. (F, G) Three‐hour post‐rPPE2/Diclofenac/PBS treatment, paw tissue samples were collected for all groups and used for tissue lysate preparation and cDNA synthesis. Next, qPCR was performed to observe transcription levels of TNF‐α (F) and IL‐6 (G). GAPDH transcript level was used as an internal control. Also, lysates were prepared from paw tissues from all groups, and an equal amount of lysate (500 μg) from each group was used to determine the levels of TNF‐α (H) and IL‐6 (I) cytokines by EIA. Same lysates were tested for MPO activity (J), and absorbance at 460 nm was measured. Data are Mean ± SEM of eight mice per group.KA subplantar injection of 5% of formalin (20 μl) was administered in the right hind paw of Balb/c mice, and an equal volume of PBS was injected in the other paw. After 3 or 24 or 48 h of formalin injection, mice were administered intraperitoneally with a single dose of either rPPE2 (3 mg/kg) or PBS (vehicle control) and after 3 h percentage inflammation in paw was recorded. Data are Mean ± SEM of six mice per group. Data information: For (D–K), unpaired *t*‐test was applied to calculate *P* values. [Colour figure can be viewed at wileyonlinelibrary.com]

Earlier we have shown that PPE2 reduces the mast cell population in a murine model of infection (Pal & Mukhopadhyay, 2021). Since the role of mast cells is prominent in tissue inflammation (Lee *et al*, 2002), we hypothesized that recombinantly purified PPE2 protein (rPPE2) can be used as a novel anti‐inflammatory drug candidate. To test our hypothesis, we used a formalin‐induced paw inflammation model system. A subplantar injection of 5% formalin (20 μl) was administered in the hind paw of Balb/c mice, and an equal volume of PBS was injected in another paw as control. After 1 h of formalin injection, mice were administered intraperitoneally with a single dose of either rPPE2 (various concentrations) or PBS (vehicle control). Paw inflammation was quantified by measuring edema/swelling for the next three consecutive hours. We observed that rPPE2 was able to reduce redness and swelling in the inflamed paw in a dose‐dependent manner, and the best effect was observed with a 3 mg/kg dose (Fig 1A and B). Diclofenac sodium at 10 mg/kg (administered intraperitoneally) was used as a standard control NSAID drug (Yin *et al*, 2016). Next, after 3 h of rPPE2 administration, paw tissues were harvested and tissue sections were stained with hematoxylin and eosin (H&E) stain. We observed marked necrotic debris and cellular infiltration in paw tissues of mice administered with PBS as vehicle control, whereas mice administered with rPPE2 showed lesser necrotic debris and cellular infiltration (Fig 1C and D). A pathology score was calculated in these groups considering swelling, redness, tissue damage, and cells infiltrated in the formalin‐injected paw, and the data reveal a reduction in the score in mice treated with rPPE2 as compared to vehicle control (Fig 1E). After 3 h of rPPE2 administration, we examined levels of pro‐inflammatory cytokines like TNF‐α and IL‐6 in paw tissues derived from all the groups by real‐time PCR (qPCR) and enzyme immunoassay (EIA). A reduction at the transcript level (Fig 1F and G), as well as at the protein level (Fig 1H and I) of both TNF‐α and IL‐6 cytokines, was observed in the paw tissues of rPPE2‐administered mice as compared to the vehicle control group. Also, there was a significant decrease in the serum levels of TNF‐α and IL‐6 cytokines (Appendix Fig S1). Mast cells are essential for neutrophil extravasation in localized inflammation and ablation of mast cell‐derived TNF‐α abrogates cellular infiltration of leukocytes (neutrophils; Dudeck *et al*, 2021). Among the immigrating leukocytes a heme‐containing enzyme, myeloperoxidase (MPO) is released mainly by neutrophils, which is regarded as a biomarker for inflammation (Loria *et al*, 2008). Therefore, next, we compared MPO activity in the paw tissue samples and observed that rPPE2‐treated mice had lesser MPO activity as compared to the vehicle control (Fig 1J). This indicates that PPE2 inhibits the recruitment of neutrophils to the inflamed tissue. When rPPE2 (3 mg/kg) was administered at 3, 24, and 48 h after formalin injection, it was found to suppress tissue inflammation at all time points (Fig 1K), suggesting a therapeutic effect of rPPE2 to treat inflammation.

**Figure EV1:**
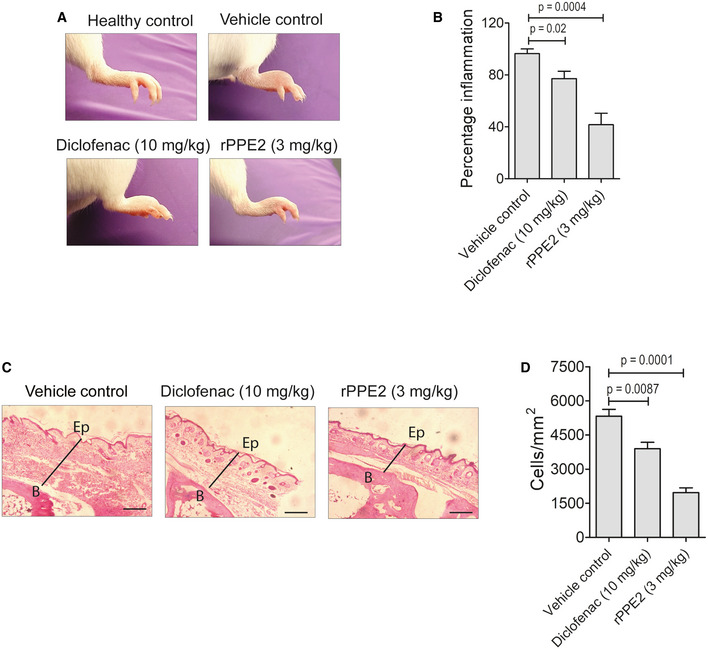
rPPE2 suppresses carrageenan‐induced inflammation in mice A subplantar injection of 1% of λ‐carrageenan (100 μl) was done in the right hind paw of Balb/c mice, and an equal volume of PBS was injected in left hind paw. After 5 h of λ‐carrageenan injection, mice were administered intraperitoneally with either PBS (vehicle control) or Diclofenac (10 mg/kg) or rPPE2 (3 mg/kg) and after 3 h, induction of inflammation was confirmed by swelling in the right hind paw using a Vernier caliper.
ARepresentative photographs of the inflamed paw.BGraphical representation of percentage inflammation in paw for all the groups.CAlso, paw sections were prepared from sacrificed mice and stained with hematoxylin and eosin and photographs of representative sections were visualized at 20× magnification (scale Bar = 100 μm). The solid line represents thickness/edema (B, bone; Ep, epidermis).DThe inflammatory cells were counted using ImageJ software and plotted as cells per mm^2^. Data information: Data shown are Mean ± SEM of eight mice per group. Unpaired *t*‐test was used to calculate *P* values.

We next investigated the anti‐inflammatory activity of rPPE2 in carrageenan‐induced paw inflammation. Carrageenan is a plant polysaccharide, and it induces inflammation by activating toll‐like receptors (TLRs). Therefore, 1% of lambda (λ)‐carrageenan was injected into the hind paw of Balb/c mice via the subplantar route. The same volume of PBS was injected into the other hind paw. After 5 h of carrageenan injection, mice were administered with either PBS (vehicle control) or rPPE2 (3 mg/kg) via intraperitoneal route, and after 3 h, swelling/edema was measured. We observed that rPPE2 suppressed redness and swelling in inflamed paws as compared to PBS (Fig EV1A and B). Next, paw tissue was harvested, and tissue sections were stained with H&E stain. We observed that similar to formalin‐induced inflammation, paw tissues from rPPE2‐treated mice showed lesser cellular infiltration and edema as compared to paw tissues from PBS‐treated mice (Fig EV1C and D).

**Figure 2:**
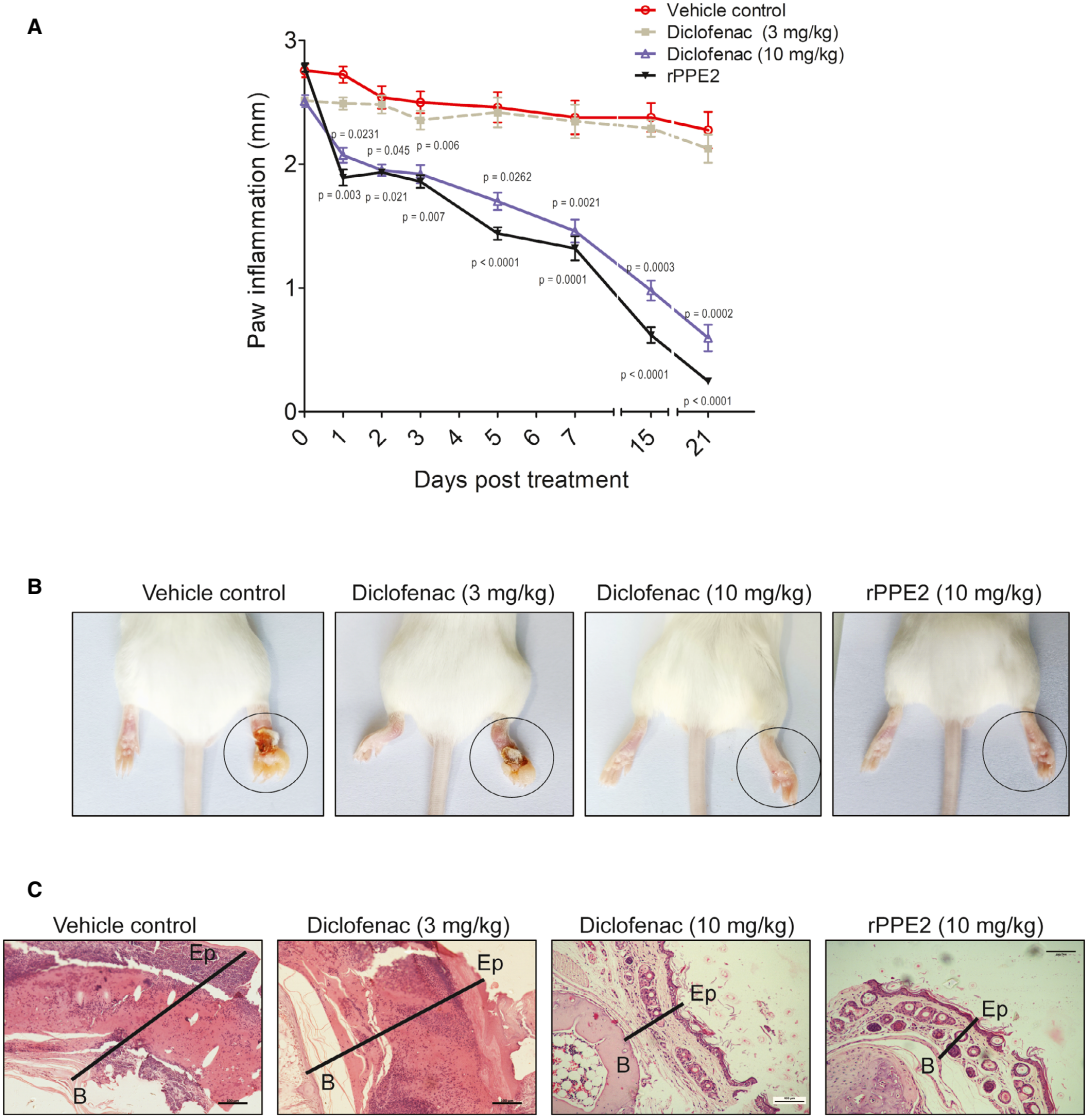
Single dose of rPPE2 prevents chronic inflammation in mice A subplantar injection of 5% of formalin (20 μl) was done in the right hind paw of Balb/c mice, and an equal volume of PBS was injected in the left hind paw. After 1 h of formalin injection, a single dose of rPPE2 (3 mg/kg) or Diclofenac (3 or 10 mg/kg) or PBS (vehicle control) was administered and paw swelling was measured for next 21 days.
AGraphical representation of inflammation (mm) in paws of mice treated with either PBS or Diclofenac or rPPE2. Data shown are Mean ± SEM of five mice. Statistical analysis was performed between groups treated with rPPE2 versus vehicle control and group treated with Diclofenac versus vehicle control, unpaired *t*‐test was applied to calculate *P* values.BThe representative photographs of inflamed paws after 21 days of post‐treatment were shown.CAfter 21 days, mice were sacrificed, and the paw tissue sections were prepared and stained with hematoxylin and eosin and photographs of representative sections were visualized at 20× magnification (scale bar = 100 μm). The solid line represents thickness/edema (B, bone; Ep, epidermis). [Colour figure can be viewed at wileyonlinelibrary.com]

Formalin induces inflammation by causing necrosis in the tissues. If left untreated, inflammation becomes chronic and causes heavy tissue damage, leading to organ immobility. We observed in the earlier section that rPPE2 (3 mg/kg) administration can suppress inflammation when administered as early as 3 h. Next, we examined the long‐term effect of rPPE2 (3 mg/kg) on the suppression of inflammation. To test this, a subplantar injection of 5% formalin (20 μl) was administered in the right hind paw of Balb/c mice and an equal volume of PBS was injected in another paw. After 1 h of formalin injection, a single dose of 3 mg/kg of rPPE2 or equal volume of PBS (vehicle control) was administered intraperitoneally and paw swelling was measured for the next 21 days. Diclofenac at both 3 and 10 mg/kg was used as positive control drug. We observed that mice administered with rPPE2 at 3 mg/kg showed a gradual reduction in paw inflammation (Fig 2A and B). Notably, mice treated with PBS showed severe damage to the paw tissues due to sustained inflammation, whereas rPPE2 administration brought down paw inflammation back to almost normal condition. Diclofenac when used at 3 mg/kg did not have any significant effect on the reduction of swelling of paw tissue; however, at a higher dose (10 mg/kg), it was able to reduce the swelling though not as efficiently as rPPE2 (Fig 2A and B). H&E staining of the paw tissue sections from the various groups revealed that rPPE2‐administered mice showed minimal tissue damage or inflammation, whereas mice administered with PBS alone (vehicle control) or 3 mg/kg of Diclofenac showed the extensive presence of necrotic tissues (Fig 2C). Our data indicate that the effect of a single dose of rPPE2 at 3 mg/kg was long‐acting and sufficient to subside inflammation and also showed better results as compared to treatment with 10 mg/kg Diclofenac. These results indicate that rPPE2 has both long‐term and short‐term anti‐inflammatory effects.

**Figure EV2:**
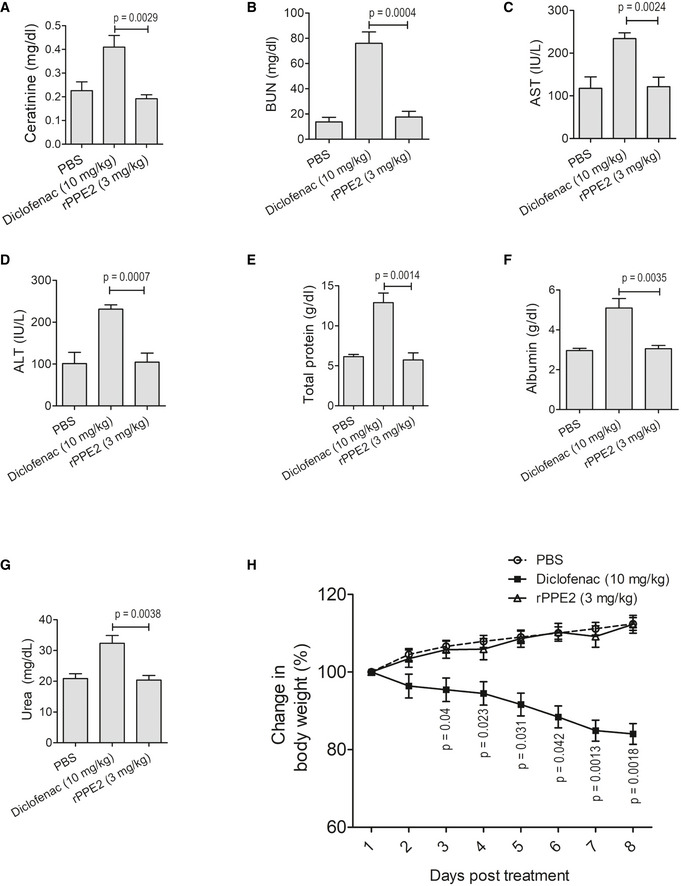
rPPE2 does not affect liver and kidney function when administered for longer duration Balb/c mice of 6–8 weeks of age were administered intraperitoneally with either Diclofenac (10 mg/kg) or rPPE2 (3 mg/kg) for 8 continuous days.
A–GOn day 9, mice were sacrificed and blood sera were analyzed for levels of creatinine (A), BUN (B), AST (C), ALT (D), Total protein (E), Albumin (F) and Urea (G). For (A–G) unpaired *t*‐test was used to calculate *P* values.HAlso, mice body weights were recorded for each group every day and change in body weight was calculated as (body weight on day X/body weight at day zero) × 100. For (H), one‐way ANOVA with Bonferroni *post hoc* test to calculate *P* values. Data information: Data shown are Mean ± SEM of five mice per group.

NSAIDs are often considered as the first line of drugs to treat inflammation (Ong *et al*, 2007). Despite their quick and effective response, usage of NSAIDs often presents various side effects and significantly increases the risks for gastrointestinal bleeding and hepatic and renal malfunctions (Davis & Robson, 2016). To test whether rPPE2 also causes organ cytotoxicity, we injected Balb/c mice with either PBS or PPE2 (3 mg/kg) via the intraperitoneal route for 8 continuous days. Since Diclofenac with only 10 mg/kg dosage was able to subside inflammation to a similar extent as that of 3 mg/kg dose of rPPE2 (Fig 2), we used Diclofenac sodium with 10 mg/kg as a standard NSAID control drug (Gupta *et al*, 2015) for toxicity studies. On day 9, mice from all the groups were sacrificed, and the blood serum was collected to check the levels of aspartate aminotransferase (AST or SGOT), alanine aminotransferase (ALT or SGPT), alkaline phosphatase (ALP) as a measure of liver function, and levels of creatinine, BUN, urea, albumin, and total protein as a measure of kidney function. It was observed that PBS‐ and rPPE2‐administered mice did not show any changes in the levels of the blood biochemical parameters (Fig EV2A–H). Also, the rPPE2‐administered group showed no change in any of the clinical features, whereas, as expected, the Diclofenac‐treated mice showed significant changes in all the tested parameters. Diclofenac‐administered mice showed ruffled fur coats, loss in body weight, and reduced activity upon stimulation. These observations indicate that rPPE2 is safe and does not impart any liver and kidney abnormality even when used for a longer duration.

### rPPE2 depletes mast cell population in the inflamed tissue

**Figure 3:**
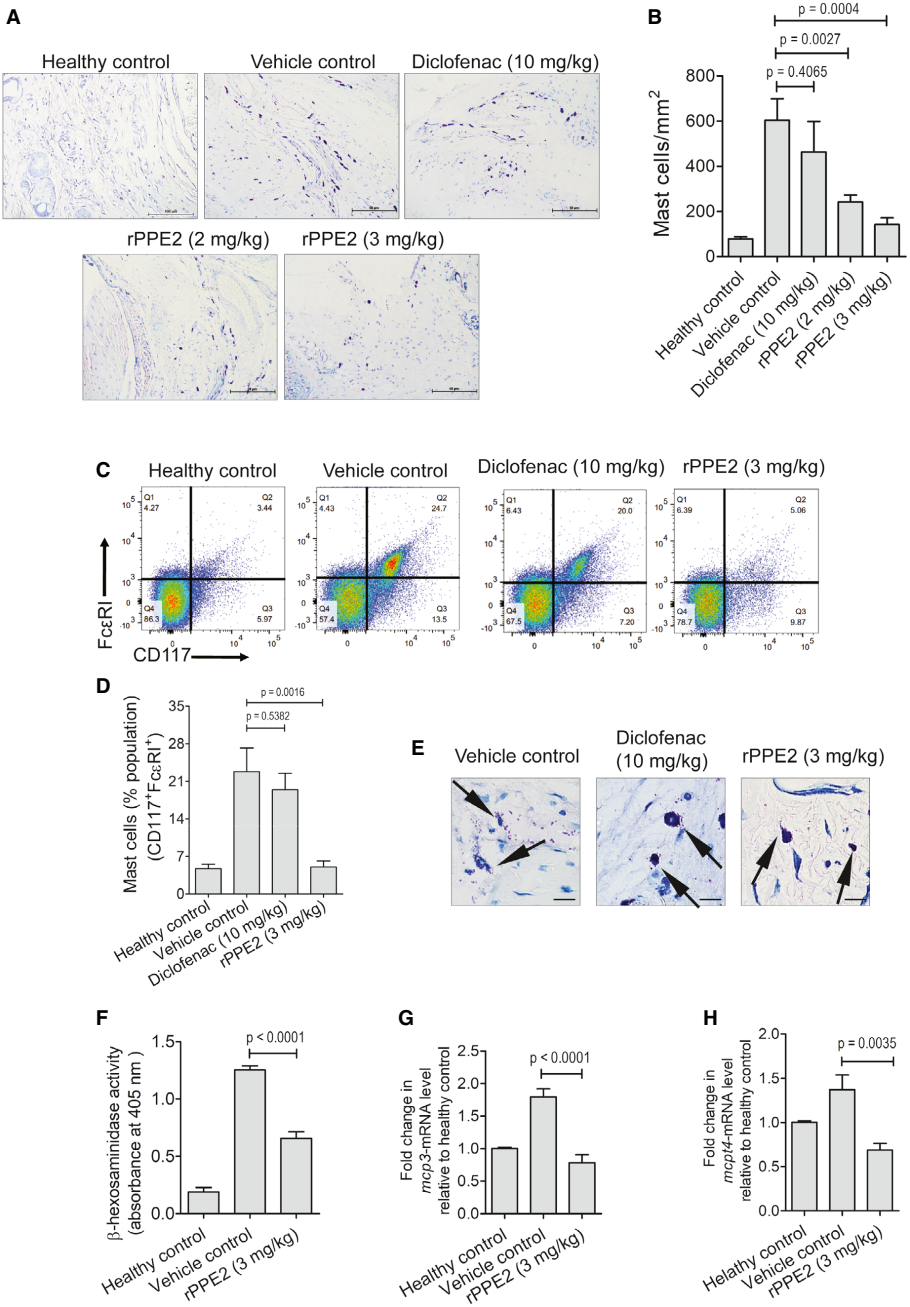
PPE2 depletes mast cell population in inflamed paw tissue A–EA subplantar injection of 5% of formalin (20 μl) was administered in the right hind paw of Balb/c mice, and an equal volume of PBS was injected in left hind paw. After 1 h of formalin injection, Balb/c mice were treated with a single dose of either PBS (vehicle control) or Diclofenac (10 mg/kg) or different concentrations of rPPE2 (2 and 3 mg/kg) via intraperitoneal route, and after 3 h of treatment, mice were sacrificed and the paw sections were prepared and stained with Toluidine blue to quantify mast cell population. (A) Photographs of representative sections were visualized at 20× magnification (scale bar = 50 μm). (B) Counting of mast cells was performed in Toluidine blue‐stained paw sections using ImageJ software and was normalized per unit area (mm^2^). (C, D) In another experiment, subplantar injection of 5% of formalin (20 μl) was carried out in the right hind paw of Balb/c mice and equal volume of PBS was injected in another paw. After 1 h of formalin injection, Balb/c mice were injected intraperitoneally with a single dose of either Diclofenac (10 mg/kg) or rPPE2 (3 mg/kg) or PBS (vehicle control), and after 3 h of treatment, mice were sacrificed and the paw tissues were collected and analyzed for mast cell population by flow cytometry using anti‐CD117 Ab and anti‐FcεRI Ab (C) and percentage population of mast cells was estimated from flow cytometry (D). (E) Photographs of representative tissue sections stained with Toluidine blue were visualized at 40× magnification to observe mast cell degranulation (scale bar = 100 μm). The arrow indicates the morphology (degranulation) of mast cells.F–H(F) Lysates from paw tissue were prepared and analyzed for β‐hexosaminidase activity at 405 nm optical density (OD). Also, paw tissues from all groups were collected and used for cDNA synthesis; qPCR was performed to observe transcription levels of MCP‐3 (G) and Mcpt4 (H). GAPDH transcript levels were used as control. Data information: Data shown are Mean ± SEM of eight mice. For (B, D, F–H), unpaired *t*‐test was applied to calculate *P* values. [Colour figure can be viewed at wileyonlinelibrary.com]

Mast cells are important for the generation of inflammatory responses. We have shown earlier that PPE2 reduces the mast cell population in a mouse model of mycobacterial infection (Pal & Mukhopadhyay, 2021). Since PPE2 inhibits inflammation, we next investigated the mast cell population in the paw tissue after rPPE2 treatment. Formalin (5%) was injected into the hind paw of Balb/c mice to induce inflammation. After 1 h of formalin injection, mice were administered with a single dose of either rPPE2 (3 mg/kg) or PBS (vehicle control) via the intraperitoneal route. Diclofenac (10 mg/kg) was used as a standard anti‐inflammatory positive control drug. After 3 h of rPPE2 treatment, mice were sacrificed and the paw tissues were harvested. Paw tissues were fixed, stained with Toluidine blue, and the mast cell population in paw in the tissues were counted for each group as described earlier (Sasaki *et al*, 2015). We observed that paw tissues from rPPE2‐treated mice showed a significant decline in the mast cell population as compared to those received PBS (vehicle control; Fig 3A and B). Diclofenac did not have any significant effect on the mast cell population (Fig 3A and B). When the cell population from the paw tissues were isolated and analyzed by flow cytometry, we observed a decrease in the mast cell population (CD117^+^, FcεRI^+^) in rPPE2‐treated mice as compared to the vehicle control group and Diclofenac‐treated mice (Fig 3C and D). This corroborates our histological observations made in Toluidine blue‐stained tissue section. We also observed that the paw tissues of rPPE2‐administered mice had reduced degranulation in mast cells as compared to the vehicle control (Fig 3E), which is also indicative of the lower inflammation in rPPE2‐treated mice.

We have shown earlier that another PE/PPE family protein PPE18 activates IL‐10 cytokine and has an anti‐inflammatory property (Nair *et al*, 2009; Ahmed *et al*, 2018). The recombinantly purified PPE18 protein (rPPE18) was shown to interact with TLR2 and inhibit LPS‐induced TNF‐α and IL‐12 production with simultaneous upregulation of IL‐10 (Nair *et al*, 2009, 2011). Therefore, we tested the ability of rPPE18 to suppress formalin‐induced paw tissue inflammation. We observed that rPPE18 (3 mg/kg) was not as effective as rPPE2 (3 mg/kg) to reduce formalin‐induced paw inflammation and significantly failed to reduce the mast cell population in the inflamed tissue (Appendix Fig S2). These observations indicate that the anti‐inflammatory property of PPE2 lies in its ability to reduce the population of mast cells in the inflamed tissue.

**Figure 4:**
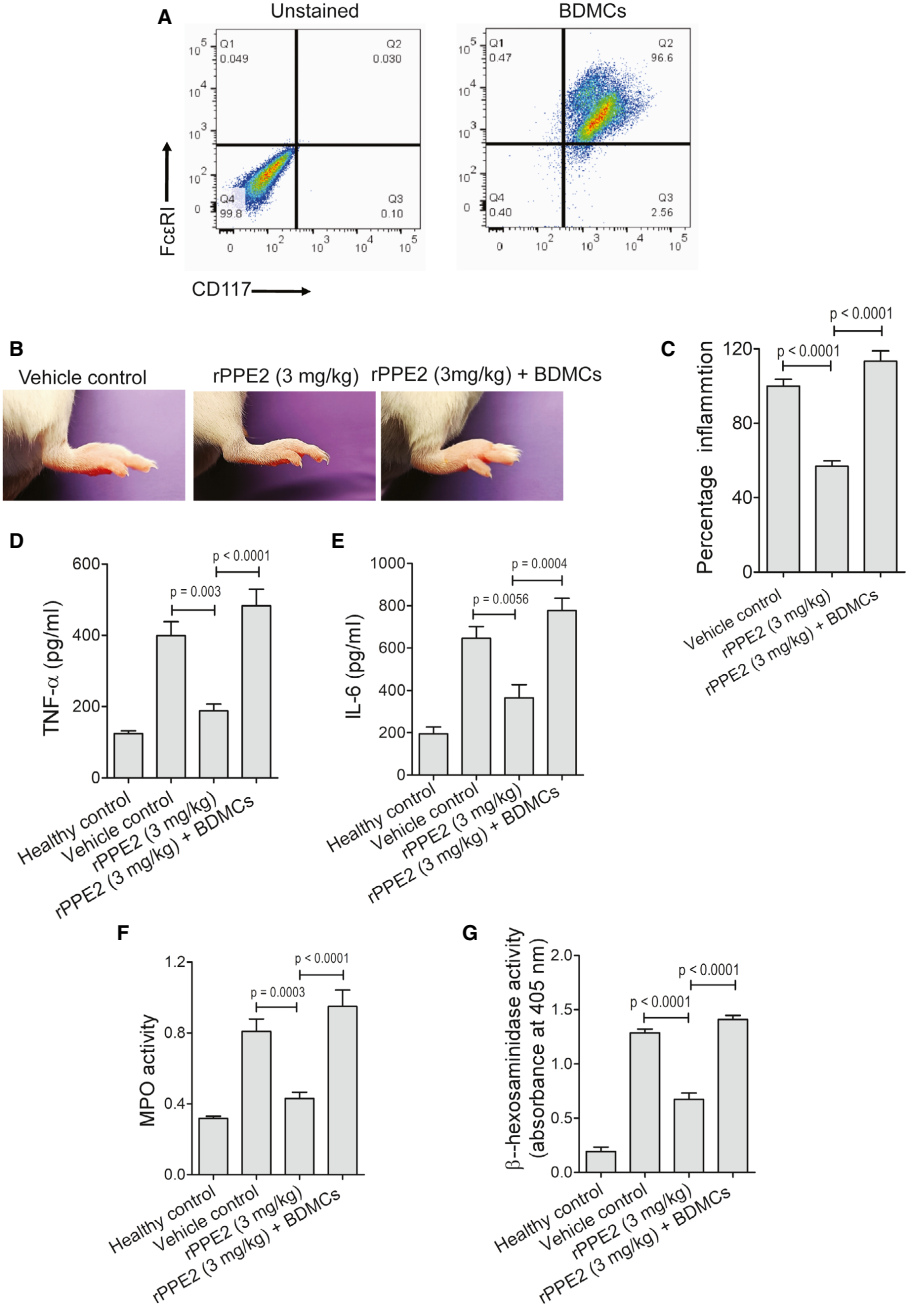
Subplantar injection of mast cells restores inflammation in rPPE2‐treated mice AMast cells were enriched from the bone marrow of the Balb/c mice in the presence of SCF and IL‐3.BA subplantar injection of 5% of formalin (20 μl) was carried out in the right hind paw of Balb/c mice, and an equal volume of PBS was injected in another paw. After 1 h of formalin injection, Balb/c mice were treated with a single dose of either Diclofenac (10 mg/kg) or rPPE2 (3 mg/kg) or PBS (vehicle control) via intraperitoneal route. After 3 h of treatment, about 1 × 10^6^ mast cells were injected via subplantar route. After 3 h of mast cell injection, the percentage inflammation was measured and the paw tissues were harvested. Representative photographs of inflamed paws after 3 h of mast cell injection.CGraphical representation of percentage inflammation in paw (paw thickness) was shown.D–GNext, tissue lysates (500 μg) were prepared and the levels of TNF‐α (D), IL‐6 (E) were measured by EIA. In the same lysate samples, MPO activity (F) and β‐hexosaminidase activity (G) were measured. Data information: Data shown are Mean ± SEM of eight mice. For (C–G), unpaired *t*‐test was applied to calculate *P* values. [Colour figure can be viewed at wileyonlinelibrary.com]

Activated mast cells secrete several mediators into the tissue microenvironment, which include mediators like histamine, serotonin β‐hexosaminidase, mast cell proteases, chemokines like MCP‐3 (CCL7), mast cell proteases like Mcpt4, kinins, etc., stored in granules as well as mediators, which are *de novo* synthesized like MCP‐1, TNF‐α etc. (Theoharides *et al*, 2012). Since we found rPPE2 administration reduced mast cell population in the inflamed tissue, we next measured β‐hexosaminidase activity and the transcript levels of MCP‐3 and Mcpt4 in these tissues. Inflammation was induced in mice hind paws using formalin, and after 1 h, rPPE2 (3 mg/kg) or PBS (vehicle control) was administered. Again after 3 h, mice were sacrificed and paw tissues were analyzed for β‐hexosaminidase activity as well as transcript levels of MCP‐3 and Mcpt4. We observed that paw tissues from rPPE2‐treated mice showed a significant reduction in β‐hexosaminidase activity (Fig 3F). Transcript levels of MCP‐3 and Mcpt4 were also found to be lower in rPPE2‐treated mice as compared to PBS‐treated mice (Fig 3G and H). These results indicated that inflammatory mediators were reduced in paw tissues of mice treated with rPPE2. To further demonstrate that PPE2 suppresses formalin‐induced tissue inflammation specifically through inhibition of mast cells, we performed a mast cell transplantation experiment, where bone marrow‐derived mast cells (BDMCs) were purified (with > 95% purity, Fig 4A) and injected into the paw of rPPE2‐treated mice via subplantar route as described elsewhere (Lee *et al*, 2002; Patel *et al*, 2016; Habuchi *et al*, 2021). When the percentage of inflammation in paw tissue was measured, an increased inflammation was observed in rPPE2‐treated mice that were injected with bone marrow‐derived mast cells when compared to rPPE2‐treated mice that did not receive any mast cells (Fig 4B and C). It appears that subplantar injection of excess BDMCs to the site of inflammation overwhelmed the protective effect of rPPE2. This indicates that rPPE2 specifically targets mast cells for its anti‐inflammatory properties. As expected, elevated levels of TNF‐α (Fig 4D) and IL‐6 (Fig 4E) cytokines, as well as increased MPO (Fig 4F) and β‐hexosaminidase activities (Fig 4G), were observed in rPPE2‐treated mice that received mast cells when compared to rPPE2‐treated mice receiving no mast cells. These studies together confirmed that PPE2 suppressed inflammation by specifically targeting the mast cells to the inflamed tissue.

**Figure EV3:**
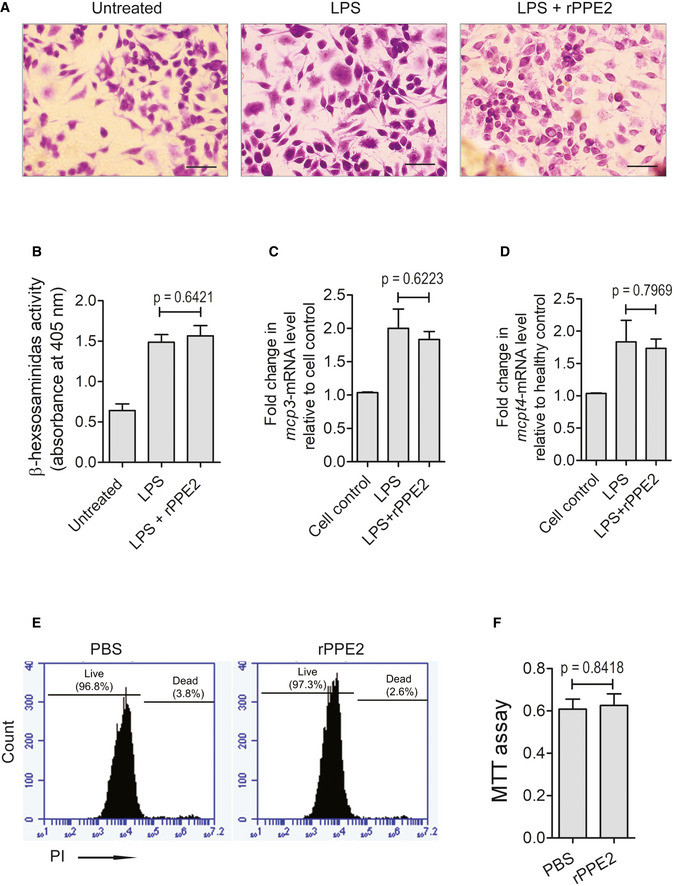
Effect of rPPE2 on mast cell activity A–DRBL‐2H3 cells were cultured in 15% fetal bovine serum (Invitrogen) in DMEM with 1× Glutamax and 1× Anti‐Anti. The cells were treated with LPS (1 μg/ml) for 3 h followed by treatment with rPPE2 (3 μg/ml). After 3 h, cells were harvested and stained with Toluidine blue. For this, cells were washed and fixed by 4% formaldehyde, washed with PBS and dehydrated using 95% ethanol followed by 100% ethanol for 30 s each. Cells were next dipped in xylene mounted on slides with mounting medium, and observed under Nikon ECLIPSE Ni‐U light upright microscope. Photographs of representative images were visualized at 10× magnification (scale bar = 50 μm) (A). For β‐hexosaminidase assay, these cells were harvested and lysed using lysis buffer (50 mM Phosphate buffer with 1% Triton X‐100) Equal amounts of cell lysates were incubated with 200 μl of 1 mM P‐nitrophenyl N‐acetyl‐beta‐D‐glucosamine (Sigma‐Aldrich, USA) dissolved in 0.05 M citrate buffer (pH 4.5). After 1 h of incubation at 37°C, absorbance was measured at 405 nm. (B). Also, these cells were used for cDNA synthesis to perform qPCR to observe transcription levels of MCP‐3 (C) and Mcpt4 (D). GAPDH transcript levels were used as an internal control.ERBL‐2H3 cells were treated with either PBS or rPPE2 (3 μg/ml). After 3 h of treatment, cells were stained with propidium iodide for 2 min and percentage of the propidium iodide‐stained population was assessed by flow cytometry.FRBL‐2H3 cells were treated with either PBS or rPPE2 (3 μg/ml) for 3 h. MTT (3‐(4,5‐dimethylthiazol‐2‐yl)‐2,5‐diphenyl tetrazolium bromide; Sigma‐Aldrich, USA) was added to the cell culture at a final concentration of 1 mg/ml for 4 h after which cells were lysed with a lysis buffer (20% SDS in 50% dimethylformamide) and absorbance was recorded at 590 nm. Data information: Data shown are Mean ± SEM of three independent experiments. (NS, no significance). For (B–D, F), Unpaired *t*‐test was used to calculate *P* values. [MCP‐3 (Forward primer 5‐GCATGGAAGTCTGTGCTGAA‐3; reverse primer 5‐CCGTTCCTACCCCTTAGGAC‐3); Mcpt4 (Forward primer 5‐GGAGCTGGAGCTGAGGAGAT‐3; 5‐reverse primer CTCCGAGGGTGACAGTGATT‐3)].

To further investigate whether rPPE2 has any direct effect on the activation of mast cells, we used a mast cell line, RBL‐2H3, and measured β‐hexosaminidase activity, and MCP‐3, Mcpt4 transcript levels in the presence or absence of rPPE2. For this, RBL‐2H3 cells were activated by lipopolysaccharide (LPS; Yang *et al*, 2012), and after 1 h, cells were treated with rPPE2 (3 μg/ml) for 3 h. Cells were harvested, stained with Toluidine blue, and observed under a light microscope. No significant visible difference in the degranulation of LPS‐activated mast cells was observed in the presence or absence of rPPE2 (Fig EV3A). Also, no significant changes in the β‐hexosaminidase activity, as well as transcript levels of MCP‐3 and Mcpt4, were observed in rPPE2‐treated cells when compared to the untreated controls (Fig EV3B–D). These results indicate that rPPE2 does not have a direct effect on mast cell activation. Also, MTT assay results showed that rPPE2 did not have any direct cytotoxic effect on the mast cells (Fig EV3E and F).

### rPPE2 is present at the site of injury and localizes to the nucleus of fibroblasts

**Figure 5:**
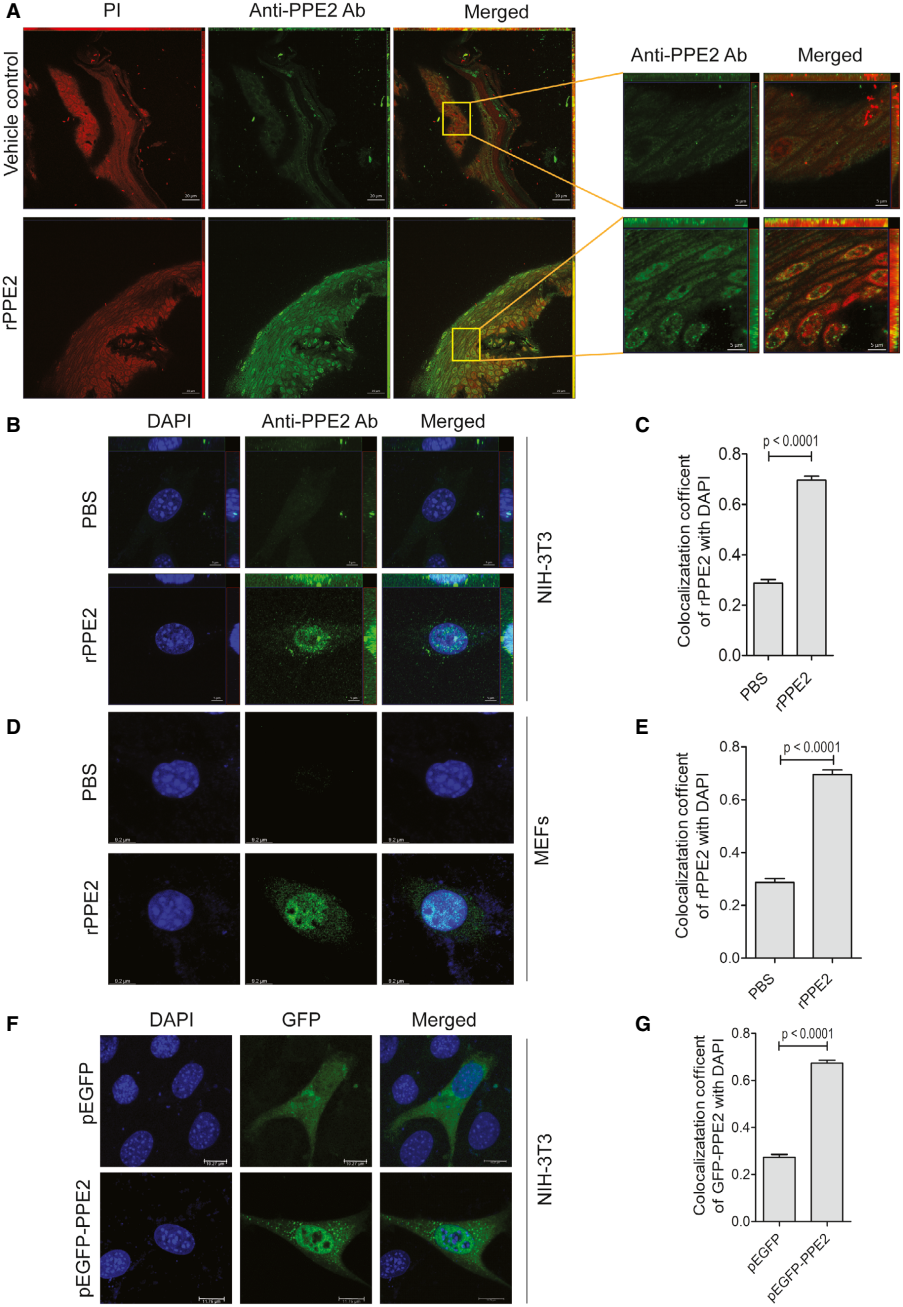
PPE2 localizes to fibroblast nucleus AA subplantar injection of 5% of formalin (20 μl) was done in the right hind paw of Balb/c mice, and an equal volume of PBS was injected in left hind paw. After 1 h of formalin injection, mice were administered with either PBS (vehicle control) or rPPE2 (3 mg/kg, single dose) via the intraperitoneal route. After 1 h of rPPE2 treatment, mice were sacrificed and the paw sections were prepared. Next, paw tissue samples were deparaffinized, subjected to antigen retrieval, and were stained with anti‐PPE2 Ab, and observed under a confocal microscope. Propidium iodide (PI) was used to stain the nucleus. (scale bar = 20 μm).B–EIn another experiment, NIH‐3T3 cells (B, C) or MEFs (D, E) were incubated with either PBS or rPPE2 (3 μg/ml). After 45 min of rPPE2 treatment, cells were fixed, permeabilized, and stained with anti‐PPE2 Ab. DAPI was used to stain the nucleus. Nuclear localization of rPPE2 in NIH‐3T3 (C) and MEFs (E) was measured by calculating the colocalization coefficient.F, G(F) NIH‐3T3 cells were transiently transfected with either pEGFP or pEGFP‐PPE2. After 24 h of transfection, cells were fixed and observed under a confocal microscope. DAPI was used to stain the nucleus. (G) Nuclear localization of rPPE2 was measured by calculating colocalization coefficient. Data information: Data represent Mean ± SEM of more than 50 cells of three independent experiments. For (C, E and G), unpaired *t*‐test was applied to calculate *P* values. [Colour figure can be viewed at wileyonlinelibrary.com]

To test the physical presence and localization of rPPE2 in the inflamed paw tissues, we injected rPPE2 (3 mg/kg) intraperitoneally in mice and after 1 h of administration of rPPE2, paw tissues were harvested and analyzed for localization of PPE2 by confocal microscopy. We observed positive staining for PPE2 (green) predominantly in the dermal and hypodermal region of the inflamed paw tissue, which are rich in fibroblast cells (Fig 5A). PBS‐injected mice were used as vehicle control. This indicates that PPE2 is present at the site of inflammation after its administration. Notably, PPE2 was seen in the nucleus (red) of the majority of the cells of the paw tissue.

We had reported earlier that PPE2 protein has a leucine zipper DNA‐binding motif and a functional nuclear localization signal (Bhat *et al*, 2017). In the present study, PPE2 localization was observed in the hypodermal and dermal regions of the paw tissue (Fig 5A), which happens to be the anatomical location of the tissue fibroblasts. Also, PPE2 was found to be localized in the nucleus (red) of the majority of the cells of the paw tissue. Therefore, next, we used NIH‐3T3 fibroblasts to observe the nuclear localization of PPE2. NIH‐3T3 fibroblasts were treated with rPPE2, and after 45 min, cells were harvested and analyzed for PPE2 localization by confocal microscopy. We observed that PPE2 was translocated to the nucleus of NIH‐3T3 fibroblast (Fig 5B and C). Similarly, PPE2 was also found to translocate into the nucleus of another fibroblast cell line, mouse embryonic fibroblasts (MEFs) indicating that PPE2 can get translocated into the nucleus of fibroblast (Fig 5D and E). Similar results were also observed when NIH‐3T3 cells were transfected with plasmid‐carrying PPE2 fused with GFP (pEGFP‐PPE2) as EGFP‐tagged PPE2 showed enhanced localization in the nucleus as compared to cells transfected with pEGFP alone (Fig 5F and G).

### rPPE2 inhibits *scf* transcription by binding to *scf* promoter

**Figure 6:**
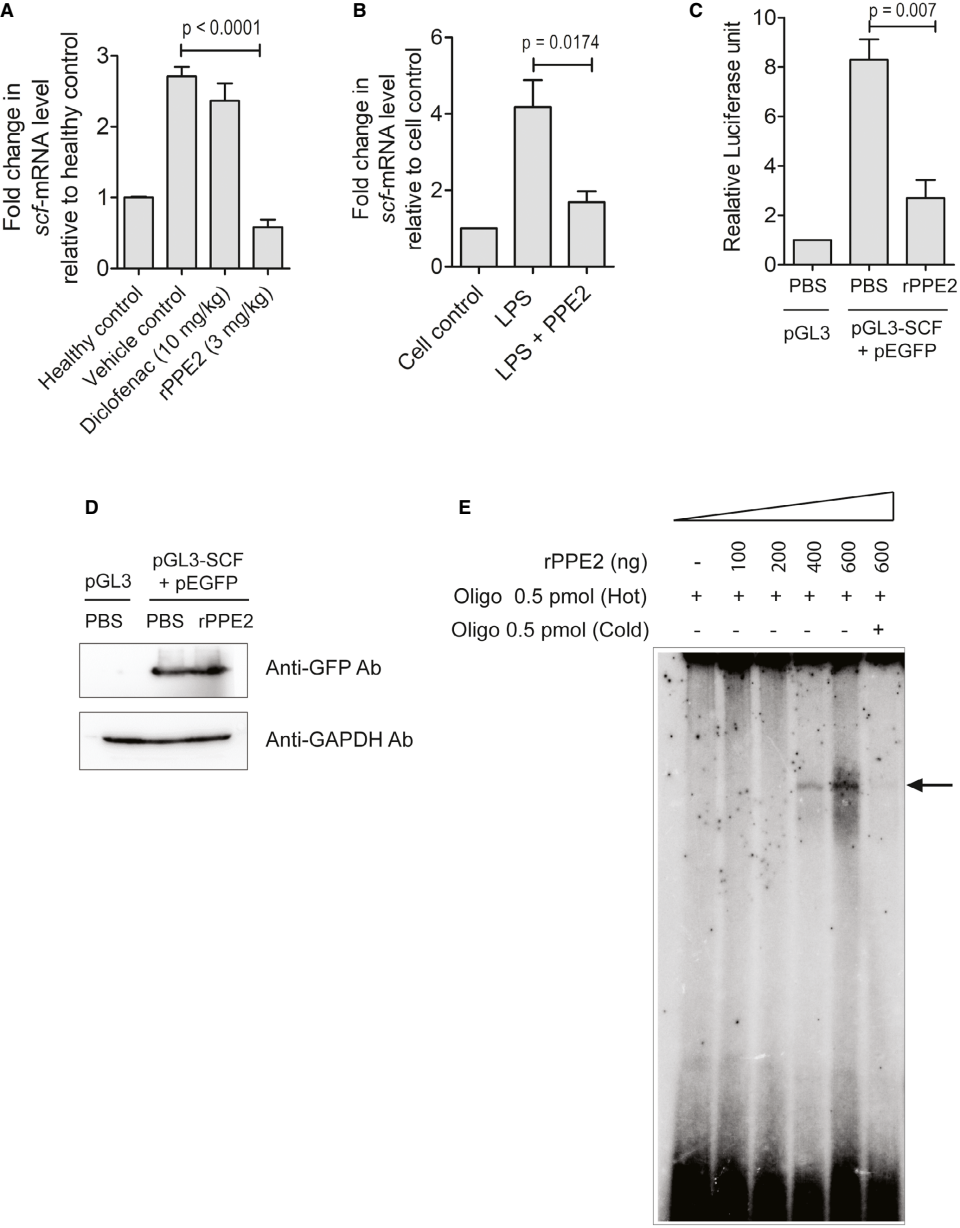
PPE2 inhibits *scf* transcription by binding to *scf* promoter AA subplantar injection of 5% of formalin (20 μl) was done in the right hind paw of Balb/c mice, and an equal volume of PBS was injected in left hind paw. After 1 h of formalin injection, mice were administered with either a single dose of Diclofenac (10 mg/kg) or rPPE2 (3 mg/kg) or PBS (vehicle control) via intraperitoneal route. After 3 h, paw tissues were harvested and used for cDNA synthesis. Next, qPCR was performed to observe transcription levels of SCF. GAPDH transcript level was used as an internal control. Data shown here are Mean ± SEM of eight mice.BNIH‐3T3 cells were activated by LPS (1 μg/ml) and after 30 min, cells were treated with rPPE2 (3 μg/ml) for 3 h. Cells were then harvested for cDNA synthesis and qPCR was performed to observe transcription levels of SCF. GAPDH transcript level was used as an internal control. Data shown are Mean ± SEM of three independent experiments.CNIH‐3T3 cells were co‐transfected with pGL3‐SCF and pEGFP. After 24 h of transfection, cells were treated with rPPE2 (3 μg/ml)/PBS. The control group received the empty vector (pGL3) alone. After 3 h of treatment with rPPE2/PBS, cells were harvested and analyzed for luciferase activity. Data shown are Mean ± SEM of three independent experiments.DLysates prepared from the same samples used in C were resolved on SDS–PAGE and checked for GFP expression by Western blotting using anti‐GFP Ab. GAPDH was used as a loading control.EVarying concentrations of rPPE2 protein were incubated with [γ‐^32^P]‐ATP oligonucleotides of 60 bp spanning SCF promoter. DNA‐protein complexes were resolved on 7% native polyacrylamide gel. In cold competition reactions, equimolar cold/unlabeled 60 bp oligonucleotides were used (arrow represents PPE2‐oligo complex). The data shown are representative of three independent experiments. Data information: For (A–C), unpaired *t*‐test was applied to calculate *P* values.

Fibroblast‐mast cell communication is important for *in situ* survival and function of mast cells (Majety *et al*, 2015). Tissue‐resident fibroblasts secrete SCF (stem cell factor), which binds to SCF receptor (SCFR) present on the mast cells surface. SCF and SCFR interaction is essential for the survival of tissue‐resident mast cells. SCF is also crucial for the migration of mast cells from peripheral blood to the tissue (Okayama & Kawakami, 2006). Since rPPE2 administration was able to reduce mast cell population, we next investigated the levels of SCF transcripts by qPCR in formalin‐injected paw tissues of mice treated with rPPE2. A significant decrease in the SCF transcripts levels was observed in the paw tissues of mice treated with rPPE2 as compared to mice received PBS (vehicle control) as well as Diclofenac (Fig 6A).

To further ascertain whether PPE2 actually inhibits SCF induction in fibroblast cells, we used NIH‐3T3 fibroblast cell line and measured the levels of SCF transcripts by qPCR. Accordingly, NIH‐3T3 fibroblasts were pre‐treated with LPS for 30 min and then either left untreated or treated with rPPE2 (3 μg/ml) for 3 h. We observed that cells co‐treated with LPS and rPPE2 had reduced levels of SCF transcript as compared to the cells treated with LPS alone (Fig 6B). These results confirm that PPE2 actually inhibits SCF in fibroblast cells.

**Figure EV4:**
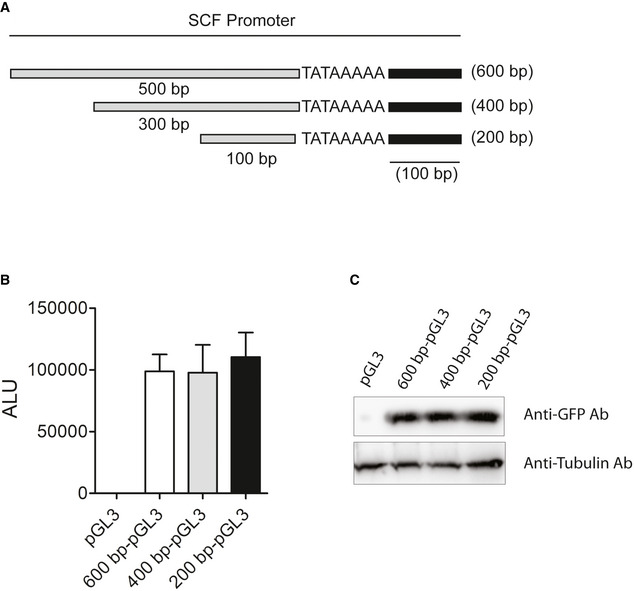
Mapping of *scf* promoter including transcription start site (TSS) AEukaryotic promoter Database (EPD) was used to predict the promoter region of the SCF gene. Three different putative promoter regions, 600 bp (−500 bp, +100 bp), 400 bp (−300 bp, +100 bp) and 200 bp (−100 bp, +100 bp) ranging over the TATAAAA (TSS) of *scf* promoter were selected for analyzing promoter activity.BThese three putative promoters were cloned in pGL3 (promoter‐less vector) and transfected in NIH‐3T3 cells along with pEGFP. After 24 h of transfection, cells were harvested and analyzed for luciferase activity. Data shown are Mean ± SEM of three independent experiments.CLysates prepared from the same samples used in experiment EV5B were resolved on SDS–PAGE and checked for GFP expression by Western blotting using anti‐GFP Ab. GAPDH was used as a loading control.

In the earlier section, we demonstrated that rPPE2 was able to localize in the nucleus of NIH‐3T3 fibroblasts and suppress SCF transcription. In our earlier study, we found that PPE2 sterically inhibits *inos* transcription by directly interacting with the GATA‐1 elements present proximal to the TATA box in the *inos* promoter (Bhat *et al*, 2017). Therefore, we next checked the promoter activity of SCF in presence of PPE2. The eukaryotic promoter database (EPD) was used to predict the promoter region of the *scf* gene (Périer *et al*, 2000). EPD predicted a 60 bp of DNA (chr10:100,015,778–100,015,837) as the putative core promoter region. To include additional regulatory elements, we selected promoter fragments of 600 bp (−500 bp, +100 bp), 400 bp (−300 bp, +100 bp), and 200 bp (−100 bp, +100 bp) surrounding the transcription start site (TSS). These promoter fragments were cloned separately into a promoter‐less vector, pGL3 (basic vector), and co‐transfected with pEGFP into NIH‐3T3 fibroblast for analyzing promoter activity via luciferase activity. We observed that all three constructs showed positive luciferase activity as compared to the vector control (Fig EV4A and B). Cell lysates from these groups were also used to check the expression levels of GFP protein by Western blotting using an anti‐GFP antibody (Ab) to ensure that all the groups had equal transfection efficiency (Fig EV4C). We proceeded with the shortest 200 bp (pGL3‐SCF) promoter region for our next experiments. To see the effect of PPE2 on SCF promoter activity, we co‐transfected NIH‐3T3 fibroblasts with pGL3‐SCF and pEGFP. The control group received the pGL3 empty vector alone. After 24 h, pGL3‐SCF transfected cells were treated with either PBS or rPPE2 (3 μg/ml) for 3 h. Cells were next harvested and analyzed for luciferase activity. We found that cells treated with rPPE2 showed reduced luciferase activity as compared to PBS control (Fig 6C). Cell lysates from these groups were used to check the levels of GFP protein by Western blotting using anti‐GFP Ab, which indicates equal transfection efficiency (Fig 6D). Taken together, these studies indicate that PPE2 inhibits the promoter activity of SCF. Since earlier we found that PPE2 binds to the *inos* promoter, we speculated a similar mechanism of transcriptional inhibition for the *scf* promoter also. Therefore, we next examined whether PPE2 physically interacts with the exact core 60 bp promoter predicted by the EPD using electrophoretic mobility shift assay (EMSA). An increasing concentration of recombinantly purified PPE2 was incubated with 60 bp radiolabeled duplex oligonucleotides encompassing the core promoter element and loaded onto the gel. We observed that rPPE2 binds to the 60 bp core promoter region in a concentration‐dependent manner (Fig 6E), indicating that rPPE2 inhibits *scf* promoter activity in a way similar to that of *inos* promoter. Therefore, we speculate that rPPE2 enters into the nucleus of the fibroblast and sterically inhibits promoter activity of SCF by directly interacting with its core promoter and hence its transcription.

### A synthetic peptide derived from PPE2 subsides paw inflammation in mice

**Figure EV5:**
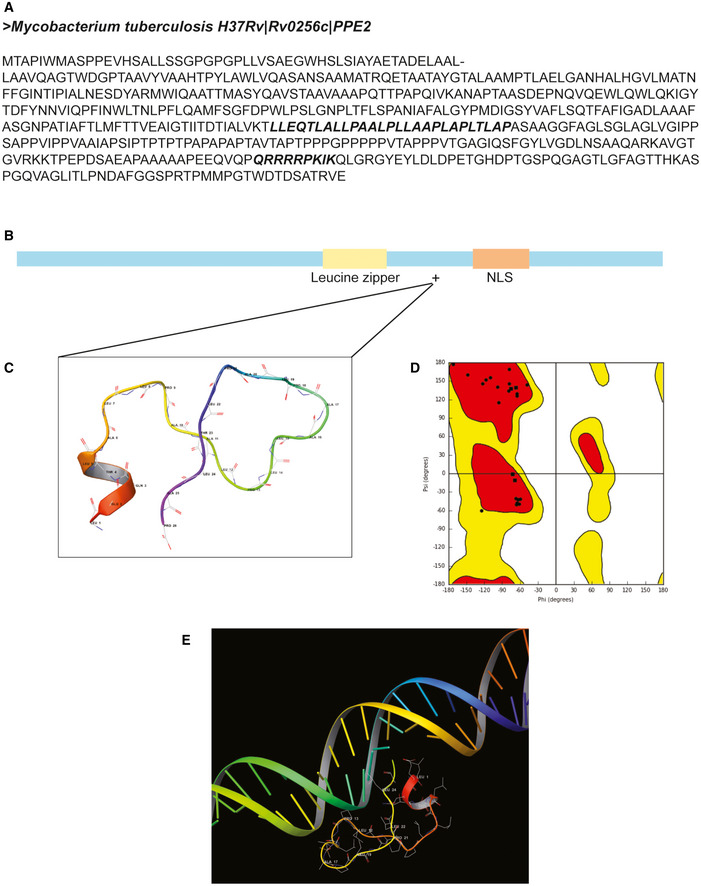
Designing of PPE2 peptide APPE2 protein of *Mycobacterium tuberculosis* possesses putative leucine zipper (DNA‐binding domain) and NLS (highlighted in bold).BA synthetic peptide of 36 amino acids is synthesized by combining leucine zipper and NLS of PPE2 (KTLLEQTLALLPAALPLLAAPLAPLTLRRRRPKIKQ; Bhat *et al*, 2017).CPeptide was modeled using MODELLER 9.23.DThe predicted model was verified by Ramachandran plot.EPeptide was docked with the EPD predicted 60 bp *scf* promoter using online HDOCK tool.

Next, we aimed to design a short peptide sequence from PPE2 for easy cellular delivery. According to the ScanProsite tool (https://prosite.expasy.org/scanprosite/), PPE2 possesses a putative leucine zipper. Based on the nuclear localization ability and DNA‐binding activity of PPE2 to *scf* promoter, we designed a synthetic peptide of 36 amino acids by combining putative leucine zipper (*KTLLEQTLALLPAALPLLAAPLAPLTL*) and nuclear localization signal (*RRRRPKIKQ*; Fig EV5A and B). To further gain insight into the DNA‐binding ability of the peptide, we performed *in silico* analysis. The peptide was modeled based on the homology using MODELLER 9.23 (Webb & Sali, 2016). The model was validated by the Ramachandran plot (Fig EV5C and D) showing that all the residues fall in the allowed regions. The modeled peptide was docked with the 60 bp of SCF promoter DNA sequences using the online tool HDOCK (Yan *et al*, 2017; Fig EV5E).

**Figure 7:**
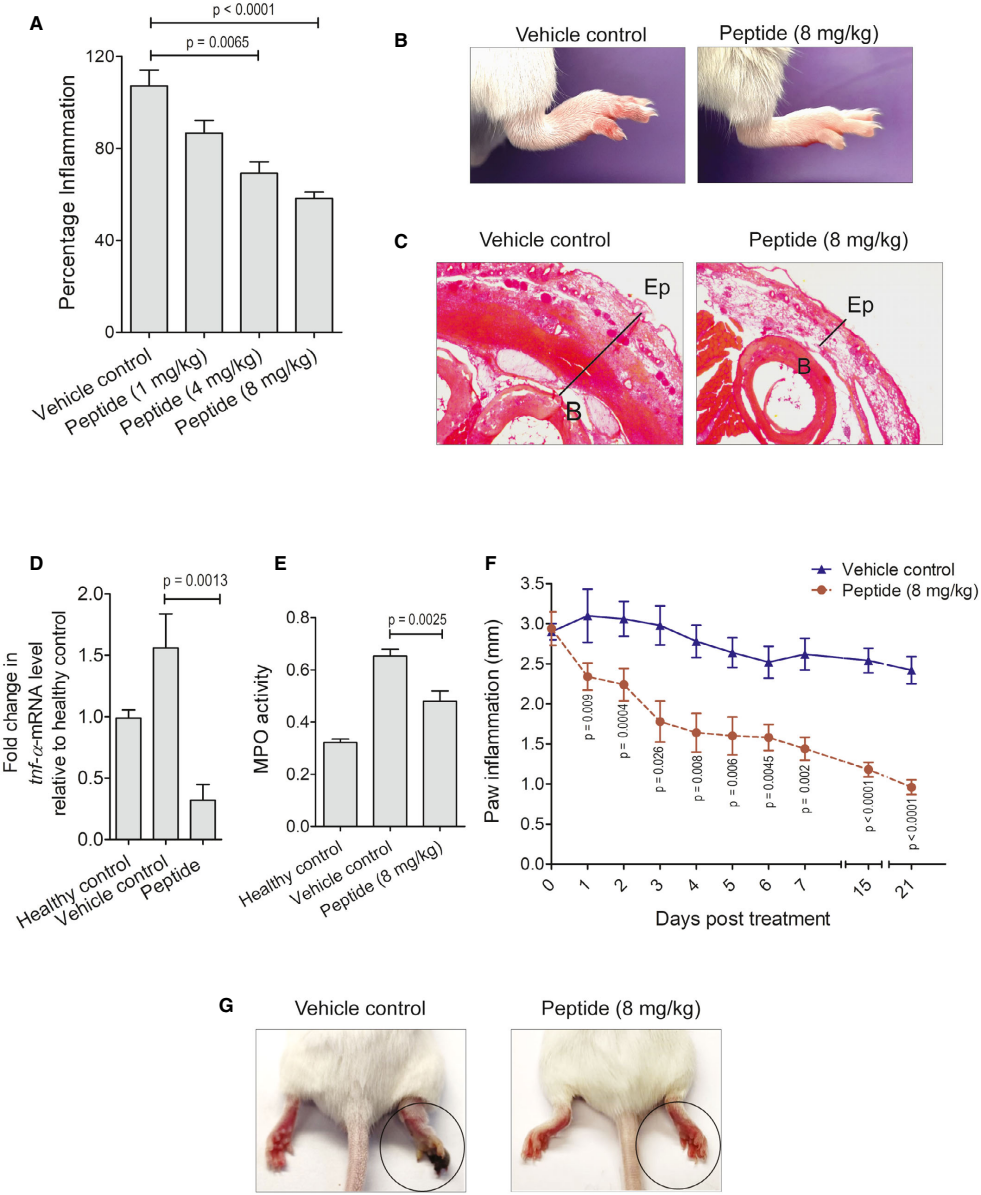
PPE2‐derived synthetic peptide subsides paw inflammation in mice A–EA subplantar injection of 5% of formalin (20 μl) was administered in the right hind paw of Balb/c mice, and an equal volume of PBS was injected in the left hind paw. After the development of edema at 1 h time point, mice were administered intraperitoneally with a single dose of either PBS or different concentrations of peptide. (A) After 3 h, mice were examined for paw thickness, and percentage inflammation was plotted. (B) Representative photographs of inflamed paws after 3 h of peptide (8 mg/kg) treatment were shown. (C) These mice were next sacrificed and the paw sections were prepared and stained with hematoxylin and eosin, and photographs of representative sections were visualized at 20× magnification (scale bar = 100 μm). The solid line represents thickness/edema (B, bone; Ep, epidermis). (D) Three hours post‐PBS/peptide treatment, paw tissue samples were collected and used for cDNA synthesis. Next, qPCR was performed to observe transcription levels of TNF‐α. GAPDH transcript level was used as an internal control. (E) Also, lysates were prepared from paw tissues and 50 μg of tissue lysate was used for testing MPO activity, and absorbance was measured at 460 nm. Data shown are Mean ± SEM of eight mice per group. For (A, D and E) unpaired *t*‐test was applied to calculate *P* values.F, GIn another experiment, a subplantar injection of 5% of formalin (20 μl) was given in the right hind paw of Balb/c mice, and an equal volume of PBS was injected in left hind paw. (F) After 1 h of formalin treatment, peptide (8 mg/kg) was administered and paw swelling was measured for the next 21 days. (G) Representative photographs of inflamed paws after 21 days of peptide treatment. Data shown here are Mean ± SEM of five mice. For (F) paw inflammation following treatment with peptide versus PBS at various days was compared using one‐way ANOVA with Bonferroni *post hoc* test. [Colour figure can be viewed at wileyonlinelibrary.com]

The anti‐inflammatory activity of PPE2‐derived synthetic peptide was assessed using paw inflammation as a model system. Balb/c mice were injected with 5% formalin in the right hind paw. After 1 h of formalin injection, a single dose of the peptide or PBS (vehicle control) was administered to the mice via the intraperitoneal route at varying concentrations (1, 4, and 8 mg/kg). Paw edema/swelling was measured after 3 h of peptide administration. We observed that similar to the recombinant PPE2 protein, the peptide was also able to reduce edema and redness as compared to the PBS (vehicle control) in a dose‐dependent manner and the best effect was observed with an 8 mg/kg dose (Fig 7A and B). Next, the paw tissues were harvested from control‐ and peptide‐ (8 mg/kg) treated groups, and tissue sections were stained with H&E dye. We observed that necrotic debris and infiltration of cells were lesser in mice treated with peptide as compared to mice treated with PBS (vehicle control; Fig 7C). Paw tissues were also harvested to check the levels of TNF‐α and MPO activity as hallmarks of inflammation. We observed a significant reduction in the mRNA levels of TNF‐α, and also MPO activity was found to be significantly low in mice treated with the peptide as compared to the vehicle control (Fig 7D and E).

The long‐term effect of the peptide on suppressing inflammation was also studied. Therefore, Balb/c mice with formalin‐induced edema were injected intraperitoneally with either PBS (vehicle control) or peptide (8 mg/kg), and paw swelling was measured for the next 21 days. Mice administered with peptide showed a gradual and significant reduction in paw inflammation (Fig 7F and G) as compared to vehicle control. Only a single dose of the peptide was found to be sufficient to subside inflammation for a longer duration. Taken together, it was concluded that the peptide derived from PPE2 also possesses anti‐inflammatory activity and can suppress inflammation in both acute and chronic inflammation conditions.

### The synthetic peptide derived from PPE2 suppresses SCF transcription and mast cell population in paw tissue

**Figure 8:**
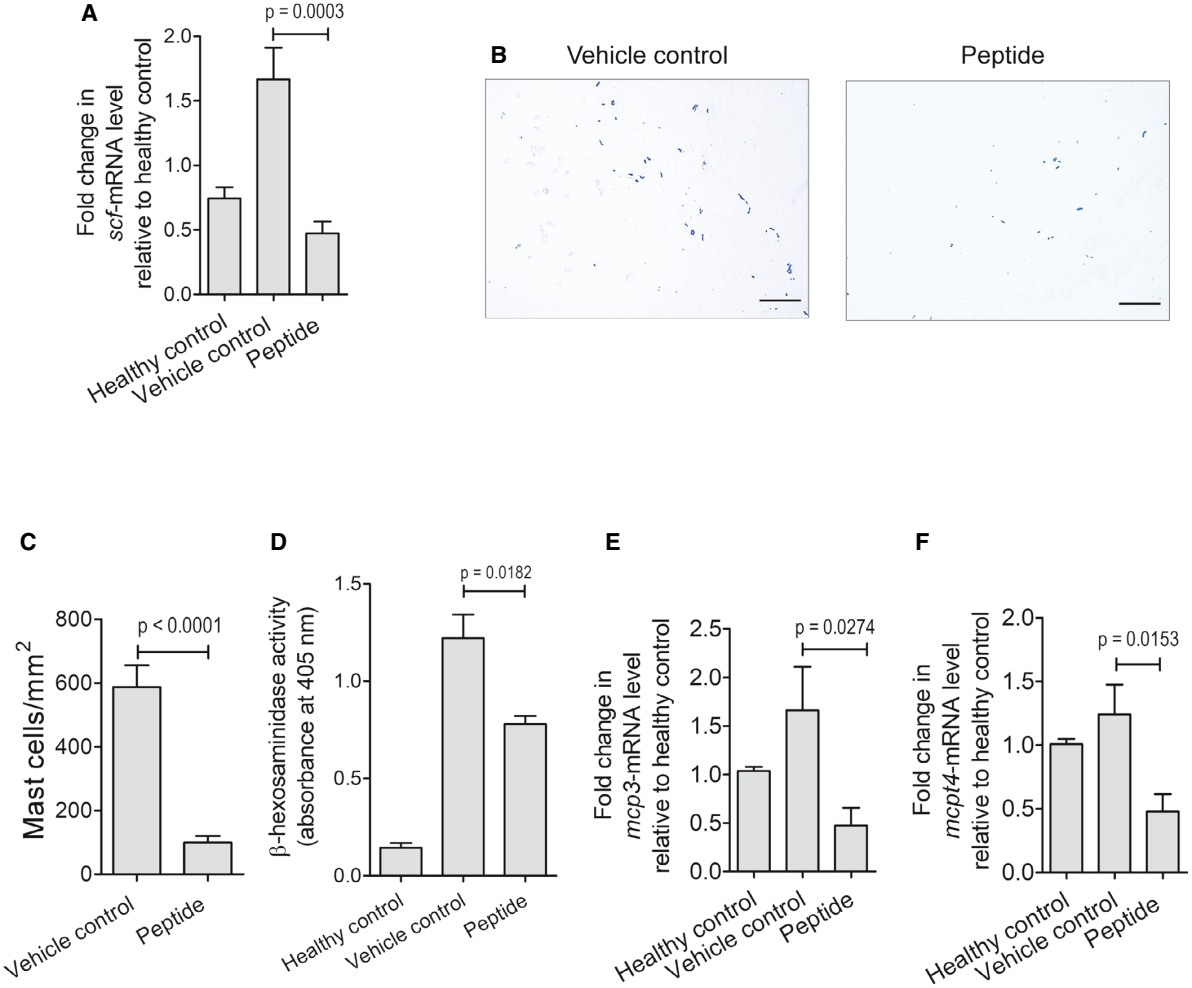
Synthetic peptide derived from PPE2 suppresses SCF transcription and mast cell population in paw tissue A subplantar injection of 5% of formalin (20 μl) was administered in the right hind paw of Balb/c mice, and an equal volume of PBS was injected in left hind paw. After induction of inflammation, Balb/c mice were treated with PBS or peptide (8 mg/kg) via the intraperitoneal route. After 3 h, paw tissues were harvested and used for cDNA synthesis. A, B(A) qPCR was performed to observe transcription levels of SCF. GAPDH transcript levels were used as an internal control. In another experiment, paw tissues from formalin‐injected mice treated with PBS/peptide were collected to prepare tissue sections. (B) Next, Toluidine blue staining was done to check the mast cell population. Photographs of representative sections were visualized at 20× magnification (scale bar = 100 μm).CCounting of mast cells was performed in Toluidine blue‐stained paw sections using ImageJ software and was normalized per unit area (mm^2^).DTissue lysate (50 μg) from each group was analyzed for β‐hexosaminidase activity and absorbance was measured at 405 nm.E, FThree‐hour post‐peptide (8 mg/kg) treatment, paw tissue samples were collected for all groups and used for cDNA synthesis. qPCR was performed to observe transcription levels of MCP‐3 (E) and Mcpt4 (F). GAPDH transcript levels were used as control. Data information: Data shown are Mean ± SEM of eight mice. For (A, C–F) unpaired *t*‐test was applied to calculate *P* values. [Colour figure can be viewed at wileyonlinelibrary.com]

Next, we studied whether the PPE2‐derived peptide can also target *scf* gene transcription in the mast cell. Therefore, 5% formalin was injected in the hind paw of Balb/c mice to induce paw inflammation in the absence or presence of 8 mg/kg peptide. After 3 h of peptide treatment, mice were sacrificed and the paw tissue samples were harvested. SCF transcript level was quantified by qPCR. We observed a significant decrease in SCF transcripts in the peptide‐treated paw tissues when compared to the paw tissues of vehicle control (Fig 8A). In Toluidine blue‐stained paw tissue sections, the mast cell population was also found to be reduced in peptide‐treated mice as compared to vehicle control (Fig 8B and C). Also, when β‐hexosaminidase activity, as well as transcript levels of MCP‐3 and Mcpt4, were measured, it was found that peptide‐treated mice had reduced β‐hexosaminidase activity and lower MCP‐3 and Mcpt4 transcript levels as compared to vehicle control (Fig 8D–F). These results indicate that the administration of PPE2‐derived synthetic peptide can also reduce the mast cell population and its mediators in the inflamed tissues.

## Discussion

Inflammation is characterized by the activation and recruitment of immune cells in response to infection and tissue injury. Under normal physiological conditions, inflammation is tightly upregulated in the presence of a threat and resolves once the threat fades away. However, an excess unregulated inflammation or inability to resolve an inflammatory response often leads to the development of diseases like atherosclerosis, cancer, and autoimmunity (Netea *et al*, 2017). Biologic therapeutics often employs engineered proteins/antibodies that target specific players in a disease process and is emerging rapidly due to their actions on selective targets and thereby have fewer side effects. The immune system, especially in the context of inflammation, has been the focus of the development of biological therapeutics over the last few decades. Several monoclonal antibody‐based therapeutics have been approved targeting TNF‐α to control inflammation in inflammatory diseases like rheumatoid arthritis and Crohn's disease (Molinelli *et al*, 2016).

Earlier we reported that a mycobacterial protein PPE2 interferes with myelopoiesis leading to the reduction in the population of myeloid cells in the peripheral blood without affecting the population of lymphoid cells (Pal & Mukhopadhyay, 2021). PPE2 appears to be a multifunctional protein that could affect the production of nitric oxide (NO) by binding to the *inos* promoter and also inhibited the production of reactive oxygen species (ROS) by destabilizing NADPH‐oxidase complex in the phagosome (Pal *et al*, 2021).

Mast cells are credited to provide a “jump start” of immune responses to the site of injury by fast recruitment of inflammatory cells because of their ability to release preformed mediators immediately as well as rapid elaboration of lipid mediators (Nigrovic & Lee, 2005). The role of the mast cells in inflammation appears to be critical as mast cell knockout mice failed to develop joint inflammation/arthritis, but susceptibility was restored when these mice were engrafted with mast cells (Lee *et al*, 2002). Since PPE2 was found to affect myeloid cell population that includes mast cells, we speculated that recombinantly purified PPE2 may be used to suppress inflammation and potentially be used as an anti‐inflammatory biologic. Therefore, in the present study, we used formalin‐induced tissue injury in the hind paw of mice as a model of inflammation and investigated the effect of PPE2 on this model. Interestingly, we found that a single dose of 3 mg/kg of rPPE2 administered through the intraperitoneal route could bring down paw edema by more than 50% within 3 h of its administration and is comparable to that of Diclofenac sodium used as a control drug. Levels of inflammation markers like TNF‐α and IL‐6 in paw tissue as well as in serum were found to be significantly low in rPPE2‐treated mice as compared to the vehicle control and comparable to mice administered with Diclofenac. Interestingly, rPPE2 was found to be effective to inhibit inflammation even when administered at 48 h post‐tissue injury indicating its ability to act on an established full‐fledged inflammation.

Continuous usage of steroidal or nonsteroidal anti‐inflammatory drugs is associated with hepatic and renal toxicities. Although NSAIDs are quick and effective, they offer limited usage due to various side effects. We administered Diclofenac sodium (10 mg/kg) every day for the same duration of 8 days, and as expected, Diclofenac has deteriorated liver and kidney functions, which manifested into clinical symptoms of ruffling of fur coats, reduced activity on stimulation, and reduced body weight. On the contrary, when administered continuously for 8 days, rPPE2 (3 mg/kg) did not show any side effects on the kidney and liver functions. This indicates that prolonged usage of rPPE2 is probably safer than Diclofenac. Additionally, proteins are molecules with a fixed structural conformation and therefore, unlike typical small‐molecule chemical drugs, proteins are highly specific and, thus, result in decreased toxicity and other adverse side effects (Bruno *et al*, 2013).

Formalin administration causes acute inflammation characterized by mast cell degranulation and swelling (Rosland *et al*, 1990). Mast cells are considered as sensors of tissue damage and are recruited to the site of tissue injury (Enoksson *et al*, 2011). Mast cells also play a role in recruiting neutrophils to the damaged tissue essential for mounting efficient innate immunity (Schramm & Thorlacius, 2004). When we compared the mast cell population in the paw tissues in all the groups, we found that a single‐dose administration of rPPE2 (3 mg/kg) significantly reduced the mast cell population and levels of mast cell‐specific mediators (β‐hexosaminidase, MCP‐3, and Mcpt4). Interestingly, rPPE2 has no direct effect on mast cell activation or viability, indicating that PPE2 possibly targets mast cell population indirectly in the inflamed tissue. Subplantar injection of mast cells in the paw tissue was able to restore the inflammation in mice treated with rPPE2, indicating that PPE2 specifically targets the mast cells for its anti‐inflammatory effect.

Earlier we reported that another mycobacterial protein, PPE18, belonging to the same PPE family, can inhibit sepsis‐induced inflammation (Ahmed *et al*, 2018). Therefore, we compared the efficacy of rPPE18 with rPPE2 in suppressing formalin‐induced paw inflammation. However, we observed that the administration of rPPE18 did not suppress inflammation as efficiently as rPPE2. Interestingly, it was found that population of mast cells to the inflamed tissue was not significantly different in the presence of rPPE18 as compared to vehicle control, unlike PPE2 where a significant decrease was observed. Interestingly, Diclofenac did not have a significant effect on the reduction of mast cell population although its ability to reduce paw inflammation was slightly less to that of rPPE2. Diclofenac is known to inhibit prostaglandin synthesis through inhibition of cyclooxygenase‐1 (COX‐1) and cyclooxygenase‐2 (COX‐2) activity (Gan, 2010; Inoue *et al*, 2013) to suppress inflammation. Therefore, the anti‐inflammatory property of PPE2 appears to be predominantly due to its ability to reduce the mast cell population at the site of inflammation.

When we examined the physical presence of rPPE2 at the site of inflammation, it was found that rPPE2 is predominantly localized in the fibroblastic cells of the dermal and hypodermal regions. Using mouse embryonic fibroblasts and NIH‐3T3 fibroblastic cell lines, nuclear localization of PPE2 was observed as PPE2 contains a functional NLS (Bhat *et al*, 2017). Since PPE2 also contains a DNA‐binding motif and is known to inhibit transcription from the *inos* promoter (Bhat *et al*, 2017), we speculated that it may interfere with some factors present in the fibroblastic cells essential for mast cell recruitment to the site of injury, which may explain a decrease in the mast cell population observed in the paw edema upon PPE2 administration. Interestingly, we found that PPE2 did not directly affect either viability or functions of the mast cells. Interaction of mast cells with the neighboring cells appears to be critical for its expansion and viability (Hogaboam *et al*, 1998). The stem cell factor (SCF) produced by the fibroblasts plays an important role for mast cell survival by suppressing apoptosis (Iemura *et al*, 1994; Jensen *et al*, 2008). The level of SCF receptor (c‐kit) and SCF is known to be upregulated in cases of Mastocytosis and mast cells associated cancers (Pittoni *et al*, 2011). SCF secreted by the fibroblasts also enhances blood to tissue migration of mast cells and their migration within the tissue (Okayama & Kawakami, 2006). Therefore, we speculated that PPE2 may affect *scf* transcription by virtue of its ability to localize into the nucleus and DNA‐binding properties. Expectedly, we found a significant reduction in the *scf* transcript both in the inflamed tissue and in NIH‐3T3 fibroblasts in the presence of rPPE2. Inhibition of functional *scf* promoter activity was further confirmed using a *scf* promoter‐driven reporter assay. Mechanism of inhibition appears to be similar to that of *inos* promoter as the minimal *scf* promoter containing a 60 bp core region around the TATA box was found to specifically interact with purified PPE2 as observed in EMSA. Therefore, PPE2‐mediated suppression of *scf* transcription appears to be the reason for the low mast cell count observed in rPPE2‐administered mouse paw tissue.

One of the major problems of protein‐based therapeutics is their tendency to trigger an unwanted immune response against themselves. One of the strategies to overcome this problem would be designing smaller peptides to avoid immune surveillance. Thus, we designed a PPE2‐derived synthetic peptide by combining its putative leucine zipper DNA‐binding motif and nuclear localization signal (NLS). Based on homology modeling and peptide‐DNA docking score, we synthesized a peptide and used it to analyze its anti‐inflammatory activity in paw inflammation. It was found that the peptide containing the NLS and DNA‐binding domain together was enough to replicate the PPE2 functions and reduce the mast cell population in a similar way. The peptides/smaller molecules are more stable, easy to chemically synthesize, and tend to have a longer shelf‐life than macromolecules. Proteins/peptides are of great therapeutic value because these biomolecules have a specific 3‐dimensional geometry that enables them to act either as enzymes, hormones, interferons, or antibodies with high competency and accuracy as compared to drug molecules. Therefore, this class of therapeutic drugs is emerging as an important class of medicines in novel therapies.

Mast cells are unique as compared to other inflammatory cells. Mast cells are the only cells that store pro‐inflammatory cytokines like TNF‐α and IL‐6 in preformed granules and release them upon activation to initiate inflammation instantly. Many FDA‐approved drugs inhibit mast cell activity by neutralizing the mast cell mediators, and most of them lower the inflammation as well (Finn & Walsh, 2013). However, the beneficial therapeutic effects of neutralization of one or a few mast cell mediators are often limited due to a lack of cell specificity (Harvima *et al*, 2014). Therefore, selective suppression of mast cells may provide better and broad‐spectrum relief. In fact, targeting SCF‐c‐Kit signaling axis has been suggested as a potential therapeutic strategy to reduce mast cell population at least in some pathological conditions (Siebenhaar *et al*, 2018). Some drugs like imatinib (Cahill *et al*, 2017), nilotinib, and dasatinib, which target the tyrosine kinase activity of c‐Kit, are being evaluated for mast cell‐driven diseases (Harvima *et al*, 2014) but suffer from off‐target effects on other tyrosine kinases beyond mast cell population.

To the best of our knowledge, in this study, we report a protein and a peptide derivative that can reduce the mast cell population by targeting *scf* transcription in fibroblasts. Also, the anti‐inflammatory effect of a single‐dose administration is long‐lasting and also essentially non‐toxic in a mouse model. Though our findings are limited to formalin‐induced paw edema, it will be interesting to examine the efficacy of PPE2 and the derived peptide in other mast cell‐driven diseases in future. Thus, PPE2 and the derived peptide may constitute an important nonsteroidal biological molecule to be used successfully in the treatment of several mast cell‐driven inflammatory diseases in humans.

## Materials and Methods

### Animals

Balb/c mice of 6–8 weeks of age were used for this study. Mice were maintained at the animal house facility of Centre for DNA Fingerprinting and Diagnostics, Hyderabad, and the experimental protocols were performed as per the guidelines of the Institutional Animal Ethics Committee (IAEC).

### Protein purification

BL21 (DE3)pLysS strain of *Escherichia coli* was transformed with a pRSET‐A vector containing *ppe2* gene as described earlier (Bhat *et al*, 2017). In brief, single transformed colonies were used to set up primary culture. The primary culture was inoculated into 1,000 ml of terrific broth in the presence of antibiotics [chloramphenicol (35 μg/ml) and ampicillin (100 μg/ml)]. The culture was grown in a shaker incubator at 37°C. At an O.D. of 0.5–0.7, protein expression was induced by adding β‐D‐1‐thiogalactopyranoside (IPTG; VWR Life Sciences, USA) to a final concentration of 1 mM and was incubated for another 4 h at 37°C. Bacterial cells were harvested by centrifugation. For protein isolation, pellets were suspended in a lysis buffer [PBS containing 5% glycerol, 0.3% sodium lauroyl sarcosine, 1 mM PMSF, protease inhibitor (Sigma‐Aldrich)] and lysed using sonication. The lysate was centrifuged at 13,800 *g* for 30 min, and the supernatant was allowed to bind with the TALON resin (Takara, USA) for 30 min. The beads were next washed with a washing buffer (PBS containing 5% glycerol and 20 mM imidazole) and eluted using elution buffer (PBS containing 5% glycerol and 200 mM imidazole). Elutes were run on an SDS–PAGE gel to assess the purity of the protein. Next, the protein was dialyzed against PBS to remove imidazole from the protein samples. Dialyzed protein was passed through a column packed with Polymyxin‐B agarose beads (Sigma‐Aldrich, USA) to remove residual LPS. The concentration of the purified protein was estimated using Micro BCA™ Protein Assay Kit (Thermo Fisher Scientific, USA) following the manufacturer's instructions.

### Formalin‐induced inflammation/tissue injury

A subplantar injection of 0.02 ml of 5% formalin (Sigma‐Aldrich, USA) was administered to the right hind paw. The same volume of phosphate buffer saline (PBS) was injected into the left hind paw as vehicle control. A single dose of rPPE2 or Diclofenac was injected intraperitoneally, and induction of inflammation was confirmed at various time points by swelling in the right hind paw using a Vernier caliper. In some experiments, formalin‐injected mice were treated intraperitoneally with a PPE2‐derived synthetic peptide, and inflammation was recorded. Based on *in silico* analysis, the synthetic peptide (*KTLLEQTLALLPAALPLLAAPLAPLTLRRRRPKIKQ*) was designed and purchased from Biotech Desk Pvt Limited, India. The peptide obtained was mass spectrometer validated and HPLC purified with > 90% purity.

### Measurement of paw thickness

Paw thickness was measured using electronic digital Vernier caliper (Aerospace, India) at various time points. Percentage inflammation was calculated using the following formula:

$$\frac{\text{Post}\;-\;\text{drug treated}\;\left(\text{right hind}\;\mathrm{paw}\;-\;\text{left hind}\;\mathrm{paw}\right)}{\mathrm{Pre}\;-\;\text{drug treated}\;\left(\text{right hind}\;\mathrm{paw}\;-\;\text{left hind}\;\mathrm{paw}\right)}\;\times 100$$

### Histopathology

Mice were sacrificed, and paw samples were collected for all the groups and were fixed in 10% neutral buffered formalin followed by decalcification in 24.4% formic acid, and 0.5 N sodium hydroxide for 5 days. Tissue samples were embedded in paraffin wax and sectioned (3 ~ 5 μm) for further staining. For Toluidine blue staining, the sections were deparaffinized in xylene followed by hydration in distilled water. Further, the sections were stained in the Toluidine blue solution [0.5% Toluidine blue in 1% NaCl (pH ‐ 2.3)] for 2–3 min. Slides were washed with distilled water, dehydrated using 100% Ethanol, and mounted on the slides for image acquisition. For hematoxylin and eosin (H&E) staining, the sections were deparaffinized and hydrated in distilled water. Further, sections were stained with hematoxylin (30 s), washed in distilled water, and counterstained with eosin (1% in distilled water for 30 s). Slides were washed, dehydrated, and mounted on the slides for imaging. Tissue section images were acquired using Nikon ECLIPSE Ni‐U light upright microscope.

Histopathological severity of tissue damage was scored in a blinded fashion. The histological severity of tissue was graded as follows: 1 = minimal inflammation, minimal cellular infiltration; 2 = mild inflammation, cellular infiltration and tissue damage; 3 = moderate inflammation, cellular infiltration and necrosis; 4 = extensive inflammation, tissue damage, and necrosis.

### Isolation of mast cells from paw tissue and analysis by flow cytometry

Cells from the paw tissues were isolated as described elsewhere (Regan‐Komito *et al*, 2020). Briefly, the skin was removed, and the paw tissue was chopped into 4–5 small pieces. Next, tissue pieces were incubated in 1 ml of RPMI‐1640 supplemented with DNase I (0.1 mg/ml, Sigma‐Aldrich, USA) and Liberase (0.3 mg/ml, Roche, USA) for 90 min at 37°C. Digested soft tissue was passed through a 70 μm strainer (BD Biosciences, USA) to obtain a single‐cell suspension. For flow cytometry, cells were washed with PBS and stained with anti‐CD117 Ab conjugated to FITC (BioLegend, USA, 0.6 μg per million cells) and anti‐FcεRI Ab conjugated to APC (BioLegend, USA, 0.25 μg per million cells) diluted in staining buffer (1% BSA [bovine serum albumin] in 1× PBS containing 0.01% sodium azide) for 1 h on ice. Cells were next analyzed for mast cell population by flow cytometry (BD LSR Fortessa; BD Biosciences).

### Mouse bone marrow‐derived mast cell culture

Balb/c mice of 6–8 weeks of age were used for isolation of mast cells as described elsewhere (Meurer *et al*, 2016). Briefly, mice were sacrificed by CO_2_ asphyxiation and the femurs were removed under sterile conditions. Next, the bone marrow was isolated via flushing the bones with a syringe using complete RPMI‐1640 medium [supplemented with 10% fetal bovine serum (Gibco, USA), 1× Glutamax (Invitrogen, USA), and 1× Anti‐Anti (Invitrogen, USA)]. The bone marrow cells were further cultured for 25 days in the presence of SCF (30 ng/ml, Peprotech, USA) and IL‐3 (20 ng/ml, Peprotech, USA). The purity of the bone marrow‐derived mast cells was confirmed by flow cytometry using anti‐CD117 Ab and anti‐FcεRI Ab.

### Mammalian cell culture

NIH‐3T3 fibroblasts (ATCC) and mouse embryo fibroblasts (MEFs) were maintained in Dulbecco's Modified Eagle Medium (DMEM) supplemented with 10% fetal bovine serum (FBS), 1× Glutamax, and 1× Anti‐Anti. Cell lines were routinely tested for mycoplasma contamination.

### Beta‐hexosaminidase assay

Beta‐hexosaminidase (β‐hexosaminidase) activity in the paw tissue was measured as described earlier (Fukuishi *et al*, 2014). Briefly, the paw tissues were homogenized in a 50 mM phosphate buffer with 1% Triton X‐100. Equal amounts of tissue lysates were incubated with 200 μl of 1 mM P‐nitrophenyl N‐acetyl‐beta‐D‐glucosamine (Sigma‐Aldrich, USA) dissolved in 0.05 M citrate buffer (pH 4.5). After 1 h of incubation at 37°C, absorbance was measured at 405 nm.

### Quantification of TNF‐α and IL‐6 in tissue lysates by EIA

Levels of TNF‐α and IL‐6 cytokines in tissue lysates were quantified by sandwich EIA using Invitrogen kit (Thermo Fisher Scientific, USA) according to the manufacturer's protocol. Absolute concentration of TNF‐α/IL‐6 cytokine was measured using a standard curve provided by the manufacturer.

### Myeloperoxidase (MPO) assay

Myeloperoxidase (MPO) assay in the tissues was performed as described earlier (Pulli *et al*, 2013) with some modifications. Briefly, paw tissues were homogenized in a 20 mM phosphate buffer (pH 7.4) with 1% Triton X‐100. Tissue lysates were incubated with 20 mM phosphate buffer containing 30 mM of O‐dianisidine dihydrochloride (Sigma‐Aldrich, USA), and 20 mM hydrogen peroxide (Sigma‐Aldrich) at 37°C for 10 min, and the absorbance was measured at 460 nm.

### Transplantation of bone marrow‐derived mast cell in the paw tissue

A subplantar injection of 0.02 ml of 5% formalin (Sigma‐Aldrich, USA) was administered to the right hind paw. The same volume of PBS was injected into the left hind paw. After 1 h, rPPE2 (3 mg/kg) was injected intraperitoneally. After 3 h of rPPE2 treatment, about 1 × 10^6^ mast cells were injected into the inflamed paw via subplantar route as described elsewhere (Patel *et al*, 2016). After 3 h of mast cell administration, inflammation in paw was observed and paw tissues were harvested for assaying MPO and β‐hexosaminidase activity as well as for measuring TNF‐α and IL‐6 cytokines by EIA.

### Transfection of NIH‐3T3 cells

NIH‐3T3 cells were transiently transfected using Lipofectamine 3000 (Invitrogen, USA) following the manufacturer's protocol. Briefly, 1 μg of plasmid and 3 μl of Lipofectamine 3000 mixture were diluted separately in 100 μl of Opti‐MEM (Invitrogen, USA) and incubated at room temperature. After 20 min of incubation, the mixture was added to NIH‐3T3 cells cultured with Opti‐MEM. After 6 h of transfection, cells were replenished with fresh complete DMEM medium, and after 24 h of transfection, cells were harvested for further experiments.

### RNA isolation, cDNA synthesis, and real‐time PCR

For RNA isolation, cells or paw tissues were homogenized in the Trizol solution (Amersham), and chloroform was added. The samples were centrifuged, and the transparent supernatant was collected. An equal amount of isopropanol was added, and the mixture was centrifuged for 30 min at 18,800 *g* (4°C). The pellets were washed twice with 70% ethanol at 18,800 *g* (4°C) for 15 min. The pellet was air‐dried and dissolved in nuclease‐free water. The RNA quantity and quality were analyzed by NanoDrop (Thermo Scientific, USA). The cDNA synthesis was carried out using Moloney Murine Leukemia Virus (MMLV) reverse transcriptase as per the manufacturer's protocol (Invitrogen, USA). Real‐time PCR was carried out with a specific set of primers (Appendix Table S1) using CFX96 Real‐time PCR system (Bio‐Rad, USA) with DyNAmo Flash SYBR GREEN qPCR master mix (Invitrogen, USA) for detection. A comparative Ct method (2^−ΔΔCt^) was used to calculate the relative change in mRNA levels using expression from untreated control as base (one fold) as described elsewhere (Livak & Schmittgen, 2001). Briefly, ΔCt is the Ct value for the gene of interest normalized to the Ct value of the respective GAPDH control. ΔΔCt values were calculated as a relative change in ΔCt of the target gene in all the groups with respect to healthy control. Fold changes in the mRNA levels were expressed as 2^−ΔΔCt^. The data were analyzed using CFX Maestro software (Bio‐Rad, USA).

### Confocal microscopy

For studying PPE2 localization, cells were treated with 3 μg/ml of rPPE2 for 45 min. Cells treated with PBS were used as control. Cells were harvested and washed with PBS and fixed using 4% paraformaldehyde. Cells were permeabilized using Triton X‐100 (0.1%) and incubated with anti‐PPE2 Ab [in‐house raised (Bhat *et al*, 2017); 1:500] for overnight at 4°C. Next, cells were washed and incubated with Alexa Fluor 488–conjugated anti‐mouse secondary Ab (Thermo Fisher Scientific, USA; 1:1,000) for 60 min. Cells were washed and mounted with a mounting medium containing DAPI (VECTASHIELD, Vector labs, USA) and observed under LSM 700 Zeiss confocal microscope (Carl Zeiss Micro‐Imaging, Germany). Cells transfected with pEGP/pEGFP‐PPE2 were harvested after 24 h, washed with PBS, and fixed using 4% paraformaldehyde. Next, cells were washed and mounted with a mounting medium containing DAPI. Cells were visualized under Leica SP8 confocal microscope (Leica Microsystems, USA).

For tissues, paraffin‐embedded sections were obtained on the pre‐coated slide (Fisher Scientific, USA). The tissue sections were deparaffinized by washing with xylene (Sigma‐Aldrich, USA) followed by hydration in gradient ethanol solution (95, 75, and 50%). Next, for antigen retrieval, sections were then boiled in 10 mM sodium citrate (pH 6) for 10 min. The sections were then washed, permeabilized with Triton X‐100 (0.1%), and stained with anti‐PPE2 Ab for overnight at 4°C followed by staining with Alexa Fluor 488–conjugated anti‐mouse secondary Ab for 60 min at room temperature. Next, sections were washed and mounted with propidium iodide (VECTASHIELD, Vector labs, USA). Images were acquired on a Zeiss LSM 700 confocal microscope equipped with 405, 488, and 555 nm lasers, and fitted with a 63×, 1.4 NA objective.

### Cloning of SCF promoter

NIH‐3T3 cells were used to isolate the genomic DNA via organic extraction. Approximately, 5 × 10^7^ cells were lysed in 1 ml of lysis buffer [10 mM Tris (pH 8.0), 10 mM EDTA, 0.5% SDS, RNase]. After lysis of cells, Proteinase‐K (1 mg/ml; Sigma‐Aldrich, USA) was added and incubated for 12–16 h at 50°C. Next, an equal volume of phenol:chloroform:isoamyl alcohol (25:24:1) was added and gently vortexed. The sample was centrifuged at 18,800 *g* at room temperature, and the aqueous phase was collected. To this aqueous phase, 0.2 volumes of 10 M Ammonium acetate and 2 volumes of ethanol were added to precipitate the genomic DNA. The pellet was washed with 70% ethanol and dissolved in nuclease‐free water. Genomic DNA concentration was checked by NanoDrop, and quality was checked by running it on 0.7% agarose gel. From isolated genomic DNA, the putative promoter regions were amplified and cloned into the pGL3 basic vector (Promega, USA) at XhoI and MluI restriction sites using a specific set of primers (Appendix Table S1). The promoter activity of these clones was checked by transfecting NIH‐3T3 cells using the Luciferase kit (Promega, USA).

### Electrophoretic mobility assay (EMSA)

For EMSA analyses, the 60‐bp nucleotide (Appendix Table S1) was used. The reverse and forward complementary oligomers were annealed by incubating at 95°C for 10 min in 100 mM NaCl and gradually cooling to room temperature. The annealed oligonucleotides were end‐labeled with [γ‐^32^P]‐ATP using T4 polynucleotide kinase (New England Bio Labs) for 1 h at room temperature. For competition studies, equimolar excess of the unlabeled oligonucleotides was incubated with the labeled probes. Varying amounts of rPPE2 were incubated with 1 ng of ^32^P–end‐labeled oligonucleotide in a binding buffer (20 mM HEPES, 0.5 mM DTT, 0.5 mM EDTA, and 5% glycerol) for 30 min at room temperature. The protein‐DNA complex was resolved on a 7% native polyacrylamide gel using running buffer (25 mM Tris, 190 mM Glycine, 1 mM EDTA, pH 8.3) at 4°C. The specificity of the binding was determined by competition with equimolar excess of unlabeled probes. After the run, the gel was dried at 80°C for 1 h and exposed to an imaging plate (Fujifilm, USA) overnight. The imaging was done using a phosphor imager (GE, health care, USA).

### Generation of peptide using homology modeling and Peptide‐DNA interaction

The putative “leucine zipper” spanning region (*KTLLEQTLALLPAALPLLAAPLAPLTL*) of PPE2 was modeled using Ba3‐cytochrome‐c oxidase (1EHK) as a template using MODELLER 9.23. Based on the DOPE and GA score, the best model was selected. The resulting model was validated using PROCHEK. Ramachandran plot for the modeled peptide showed all the residues falling in the favorable and allowed regions. Next, modeled peptide structure was docked with the SCF promoter DNA sequence (predicted by EPD database) using HDOCK. The docked structure was further visualized by PyMOL.

### Statistical analysis

GraphPad Prism software, version 5.0, was used for determining the significance of the difference in the mean between the samples. Student's *t*‐test was performed to calculate the significance between two samples. For multiple group comparison, one‐way ANOVA with Bonferroni *post hoc* test was performed. *P* < 0.05 was considered to be significant.

## Data availability

This study has no data that require deposition in a public database.

## Author contributions

**Ravi Pal:** Data curation; formal analysis; validation; investigation; visualization; writing—original draft. **Madhu Babu Battu:** Conceptualization. **Sangita Mukhopadhyay:** Conceptualization; formal analysis; supervision; funding acquisition; project administration; writing—review and editing.

In addition to the CRediT author contributions listed above, the contributions in detail are:

RP and SM conceived, designed the experiments, and analyzed the data. RP performed all the experiments. MBB helped in mice inflammation experiments. RP and SM wrote the manuscript. SM corrected and edited the manuscript.

## Disclosure and competing interests statement

A patent application based on the results described in this paper is being filed by the Centre for DNA Fingerprinting and Diagnostics, in which SM and RP and MBB are listed as inventors.

## Supporting information




Appendix

Expanded View Figures PDF
