# Supplementary material for: Therapeutic application of PPE2 protein of Mycobacterium tuberculosis in inhibiting tissue inflammation
Source: EMBO Mol Med. 2022 Jul 11;14(9):e14891. doi: 10.15252/emmm.202114891 (PMC9449591; doi:10.15252/emmm.202114891)
Supplement: Supplementary file 1 — Appendix [file EMMM-14-e14891-s002.pdf]

Therapeutic application of PPE2 protein of *Mycobacterium tuberculosis* in inhibiting tissue inflammation

Ravi Pal[*,#] Madhu Babu Battu[*] and Sangita Mukhopadhyay[*,¶]

[*]Laboratory of Molecular Cell Biology, Center for DNA Fingerprinting and Diagnostics (CDFD), Inner Ring Road, Uppal, Hyderabad - 500039, Telangana, India

[#]Graduate Studies, Manipal Academy of Higher Education, Manipal - 576104, Karnataka, India

[¶]**To whom correspondence should be addressed:** Laboratory of Molecular Cell Biology, Center for DNA Fingerprinting and Diagnostics (CDFD), Inner Ring Road, Uppal, Hyderabad - 500039, Telangana, India. Tel: +91-40-27216134; E-mail: sangita@cdfd.org.in

Table of contents

Appendix Table S1: Table 1. List of oligonucleotides used in the study.

Appendix Figure S1. Serum levels of TNF-α and IL-6 cytokines are reduced in mice treated with rPPE2.

Appendix Figure S2. rPPE18 is not as effective as rPPE2 to reduce formalin-induced paw inflammation.

Table S1: List of oligonucleotides used in the study

Real-time PCR (qPCR)

**TNF-α**	Forward primer- 5’ GGTGCCTATGTCTCAGCCTCTT 3’<br>Reverse Primer- 5’ GCCATAGAACTGATGAGAGGGAG 3’
**IL-6**	Forward primer- 5’ TACCACTTCACAAGTCGGAGGC 3’<br>Reverse Primer- 5’ CTGCAAGTGCATCATCGTTGTTC 3’
**MCP-3**	Forward primer- 5’ CAGAAGGATCACCAGTAGTCGG 3’<br>Reverse Primer- 5’ ATAGCCTCCTCGACCCACTTCT 3’
**Mcpt-4**	Forward primer- 5’ CGACTATAACCTCCAGGTCTGC 3’<br>Reverse Primer- 5’ GAGGAGATTCGGGTGAAGACTG 3’
**SCF**	Forward primer- 5’ TCCAAGAAAATCGCTCCGGG 3’<br>Reverse Primer - 5’ GCAAGTGATAATCCAAGTCGGG 3’
**GAPDH**	Forward primer- 5’ CATCACTGCCACCCAGAAGACTG 3’<br>Reverse Primer- 5’ ATGCCAGTGAGCTTCCCGTTCAG 3’

Molecular Cloning

**SCF-600 bp promoter**	Forward Primer- 5’ CGCGTGTAGGGGAAAAGAACCAAGTGAAGTCTATCCGAC 3’<br>Reverse Primer- 5’ ATCTCGAGTCTTCTAAGGAAAGGCAGCGCTGCGATC 3’
**SCF-400 bp promoter**	Forward primer- 5’ ATACGCGTAGACAGGGGACGCACCAGGCTCGAT 3’<br>Reverse Primer- 5’ ATCTCGAGTCTTCATAAGGAAAGGCAGCGCTGCGAT 3’
**SCF-200 bp promoter**	Forward Primer- 5’ ATACGCGTGCCCTTCAGCGCGCTCCCGG 3’<br>Reverse Primer- 5’ ATCTCGAGCGGCTGCAGATAGTCCCAGCATTGGGT 3’

EMSA oligonucleotide

**EPD predicted *scf* promoter**	5’CGCGTGCGGGCGGGAGGGAGCTGTATAAAAAGCGCTGGCGGCGCAGCAGCCGGGGCTTCATTTGCG 3’

Appendix Figure S1

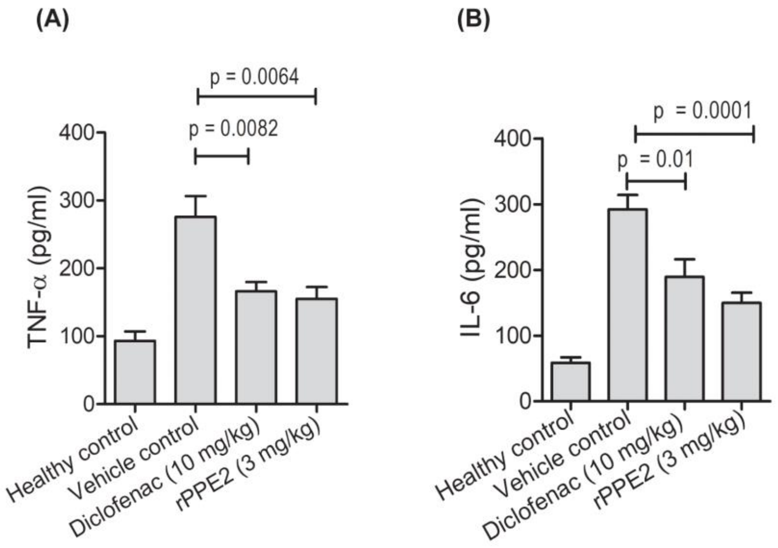

Appendix Figure S1. Serum levels of TNF-α and IL-6 cytokines are reduced in mice treated with rPPE2. A subplantar injection of 5% of formalin (20 µl) was administered in the right hind paw of Balb/c mice and an equal volume of PBS was injected in the left hind paw. After 1 hour of development of edema, mice were administered intraperitoneally with a single dose of either Diclofenac (10 mg/kg) or rPPE2 (3 mg/kg) or PBS (vehicle control). After 3 hours of treatment, mice were sacrificed and the whole blood sera were collected. The levels of TNF-α (**A**) and IL-6 (**B**) cytokines in blood sera were measured by EIA. Data shown are Mean ± SEM of 8 mice per group. Unpaired *t*-test was applied to calculate p values.

Appendix Figure S2

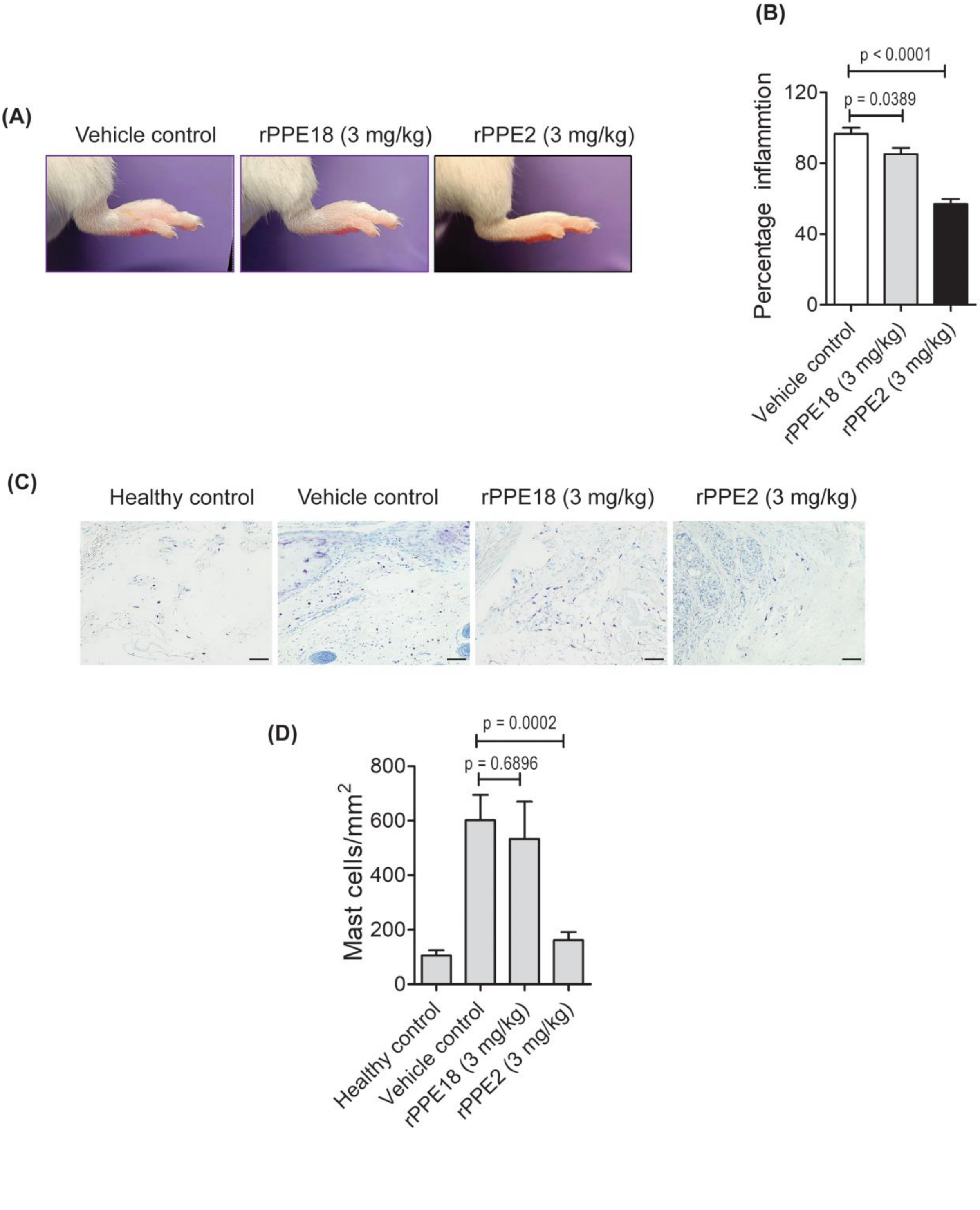

Appendix Figure S2. rPPE18 is not as effective as rPPE2 to reduce formalin-induced paw inflammation. Recombinant PPE18 (rPPE18) was purified using metal affinity chromatography. Briefly, 6X-histidine–tagged *ppe18* cloned in pRSETa, was overexpressed in *E. coli* BL21 (DE3) cells under IPTG inducible promoter. After induction, bacterial cells were pelleted down, suspended and lysed in PBS (pH 7.4) containing 0.3 mg/ml lysozyme (Sigma-Aldrich) and 1 mM PMSF (Sigma-Aldrich) and 1% sodium lauryl sarcosine (Sigma-

Aldrich). Bacterial lysates were incubated with TALON (Clontech Laboratories, Mountain View, CA) beads. rPPE18-bound TALON beads were washed with 20 mM imidazole (Sigma-Aldrich) and then eluted with 200 mM imidazole and the purified protein was collected. (**A-D**) A subplantar injection of 5% formalin (20 µl) was administered in the right hind paw of Balb/c mice and an equal volume of PBS was injected in the left hind paw. After 1 hour of development of edema, mice were administered intraperitoneally with a single dose of either rPPE18 (3 mg/kg) or rPPE2 (3 mg/kg) or PBS (vehicle control). After 3 hours of treatment, the percentage of inflammation was calculated. (**A**) Representative photographs of inflamed paws are shown after 3 hours of treatment. (**B**) Graphical representation of percentage inflammation in paw (paw thickness) was shown. (**C**-**D**) After three hours of treatment, mice were sacrificed and the paw sections were prepared and stained with Toluidine blue to check mast cell population (scale bar = 100 µm). Photographs of representative sections were visualized at 20X magnification (**C**). Counting of mast cells was performed in Toluidine blue stained paw sections using ImageJ software and was normalized per unit area (mm^2) (**D**). Data shown are Mean ± SEM of 5 mice. Unpaired *t*-test was applied to calculate p values.
