# Supplementary material for: Therapeutic application of PPE2 protein of Mycobacterium tuberculosis in inhibiting tissue inflammation
Source: EMBO Mol Med. 2022 Jul 11;14(9):e14891. doi: 10.15252/emmm.202114891 (PMC9449591; doi:10.15252/emmm.202114891)
Supplement: Supplementary file 2 — Expanded View Figures PDF [file EMMM-14-e14891-s001.pdf]

Expanded View Figures

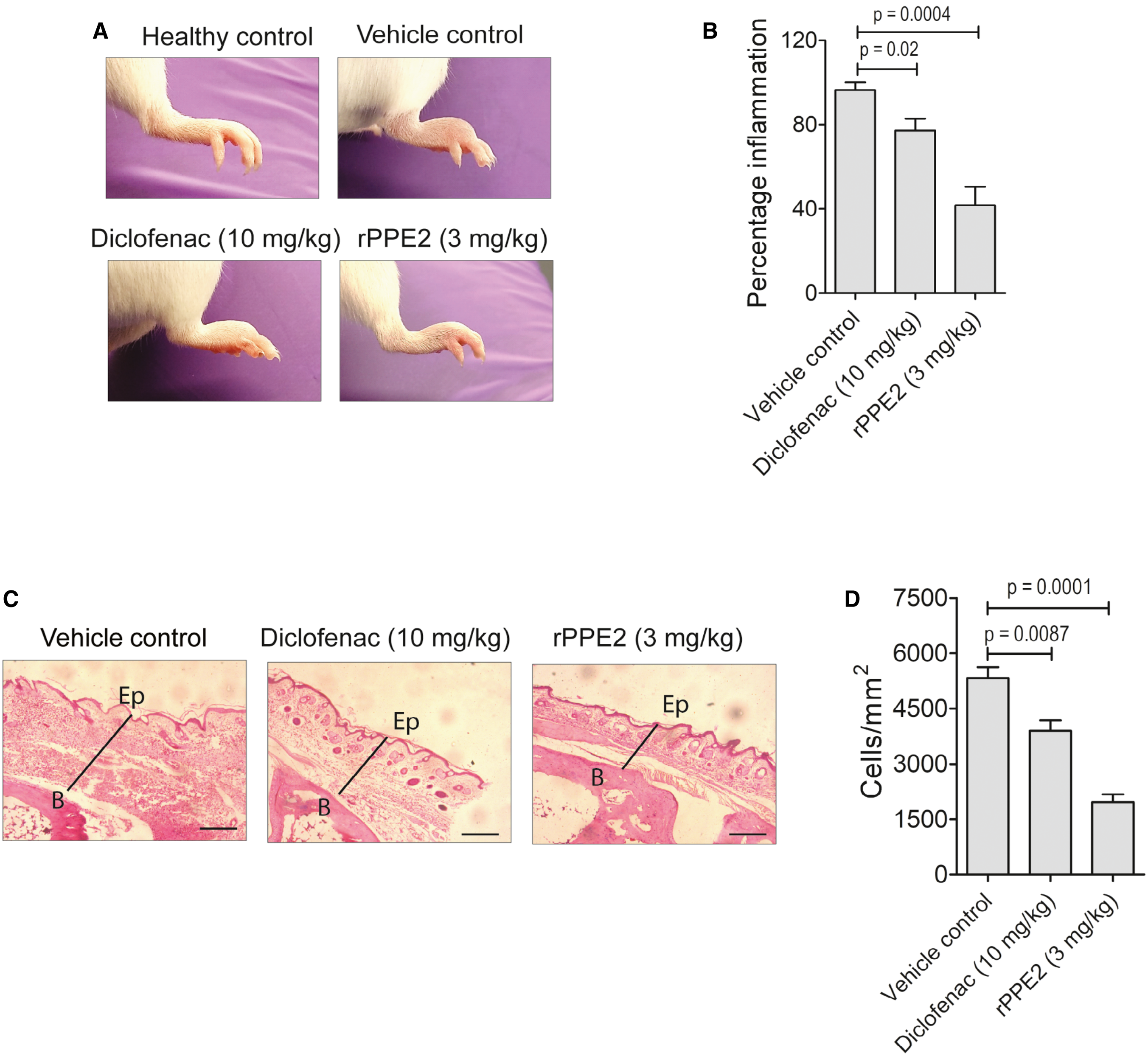

Figure EV1. rPPE2 suppresses carrageenan-induced inflammation in mice.

A subplantar injection of 1% of λ-carrageenan (100 μl) was done in the right hind paw of Balb/c mice, and an equal volume of PBS was injected in left hind paw. After 5 h of λ-carrageenan injection, mice were administered intraperitoneally with either PBS (vehicle control) or Diclofenac (10 mg/kg) or rPPE2 (3 mg/kg) and after 3 h, induction of inflammation was confirmed by swelling in the right hind paw using a Vernier caliper.

A Representative photographs of the inflamed paw.
B Graphical representation of percentage inflammation in paw for all the groups.
C Also, paw sections were prepared from sacrificed mice and stained with hematoxylin and eosin and photographs of representative sections were visualized at 20× magnification (scale Bar = 100 μm). The solid line represents thickness/edema (B, bone; Ep, epidermis).
D The inflammatory cells were counted using ImageJ software and plotted as cells per mm^2.

Data information: Data shown are Mean ± SEM of eight mice per group. Unpaired *t*-test was used to calculate *P* values.

© 2022 The Authors

Figure EV2. rPPE2 does not affect liver and kidney function when administered for longer duration.

Balb/c mice of 6–8 weeks of age were administered intraperitoneally with either Diclofenac (10 mg/kg) or rPPE2 (3 mg/kg) for 8 continuous days.

A–G On day 9, mice were sacrificed and blood sera were analyzed for levels of creatinine (A), BUN (B), AST (C), ALT (D), Total protein (E), Albumin (F) and Urea (G). For (A–G) unpaired *t*-test was used to calculate *P* values.

H Also, mice body weights were recorded for each group every day and change in body weight was calculated as (body weight on day X/body weight at day zero) × 100. For (H), one-way ANOVA with Bonferroni *post hoc* test to calculate *P* values.

Data information: Data shown are Mean ± SEM of five mice per group.

© 2022 The Authors

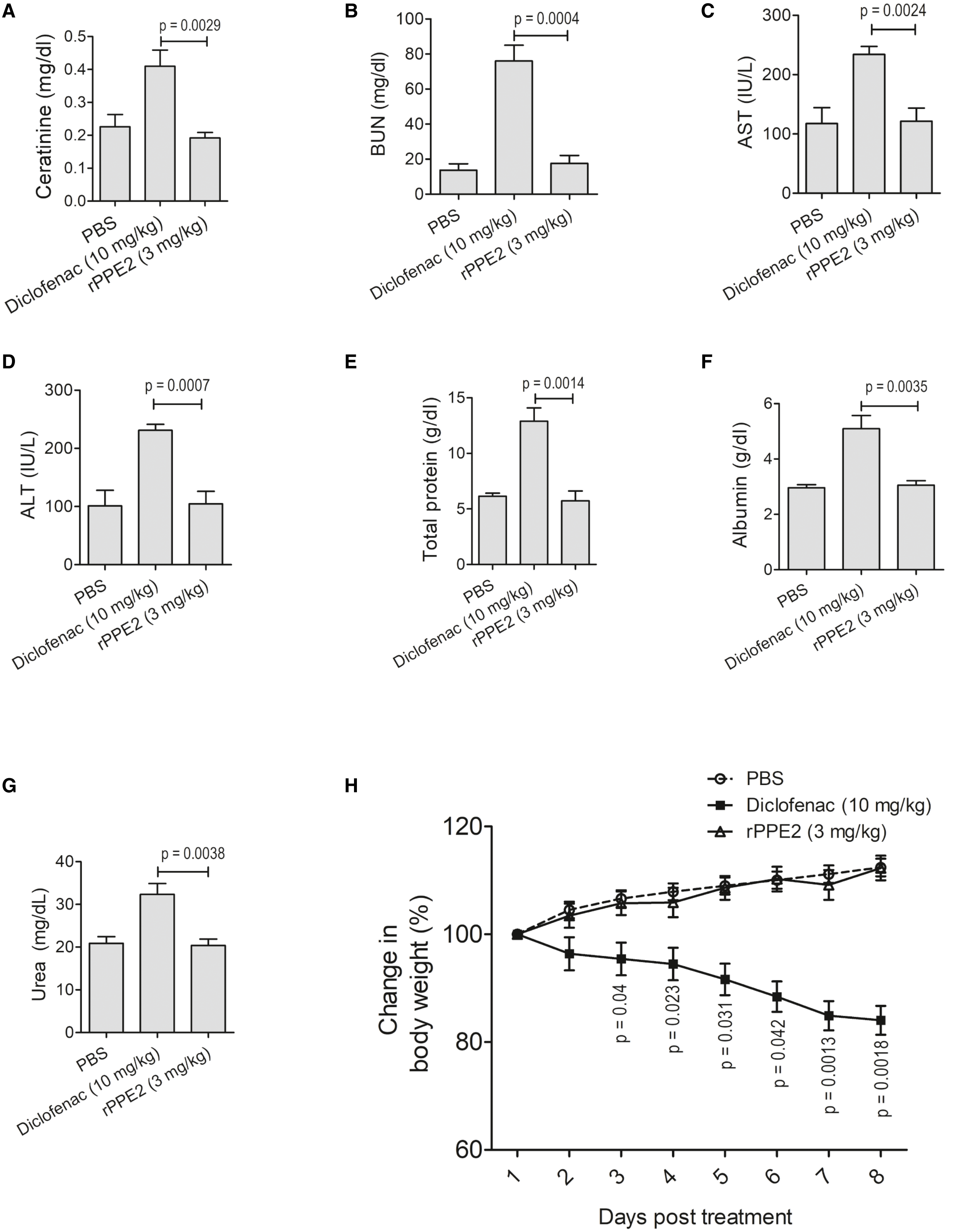

Figure EV2.

© 2022 The Authors

Figure EV3. Effect of rPPE2 on mast cell activity.

A–D RBL-2H3 cells were cultured in 15% fetal bovine serum (Invitrogen) in DMEM with 1× Glutamax and 1× Anti-Anti. The cells were treated with LPS (1 μg/ml) for 3 h followed by treatment with rPPE2 (3 μg/ml). After 3 h, cells were harvested and stained with Toluidine blue. For this, cells were washed and fixed by 4% formaldehyde, washed with PBS and dehydrated using 95% ethanol followed by 100% ethanol for 30 s each. Cells were next dipped in xylene mounted on slides with mounting medium, and observed under Nikon ECLIPSE Ni-U light upright microscope. Photographs of representative images were visualized at 10× magnification (scale bar = 50 μm) (A). For β-hexosaminidase assay, these cells were harvested and lysed using lysis buffer (50 mM Phosphate buffer with 1% Triton X-100) Equal amounts of cell lysates were incubated with 200 μl of 1 mM P-nitrophenyl N-acetyl-beta-D-glucosamine (Sigma-Aldrich, USA) dissolved in 0.05 M citrate buffer (pH 4.5). After 1 h of incubation at 37°C, absorbance was measured at 405 nm. (B). Also, these cells were used for cDNA synthesis to perform qPCR to observe transcription levels of MCP-3 (C) and Mcpt4 (D). GAPDH transcript levels were used as an internal control.

E RBL-2H3 cells were treated with either PBS or rPPE2 (3 μg/ml). After 3 h of treatment, cells were stained with propidium iodide for 2 min and percentage of the propidium iodide-stained population was assessed by flow cytometry.

F RBL-2H3 cells were treated with either PBS or rPPE2 (3 μg/ml) for 3 h. MTT (3-(4,5-dimethylthiazol-2-yl)-2,5-diphenyl tetrazolium bromide; Sigma-Aldrich, USA) was added to the cell culture at a final concentration of 1 mg/ml for 4 h after which cells were lysed with a lysis buffer (20% SDS in 50% dimethylformamide) and absorbance was recorded at 590 nm.

Data information: Data shown are Mean ± SEM of three independent experiments. (NS, no significance). For (B–D, F), Unpaired *t*-test was used to calculate *P* values. [MCP-3 (Forward primer 5-GCATGGAAGTCTGTGCTGAA-3; reverse primer 5-CCGTTCCTACCCCTTAGGAC-3); Mcpt4 (Forward primer 5-GGAGCTGGAGCTGAGGAGAT-3; 5-reverse primer CTCCGAGGGTGACAGTGATT-3)].

© 2022 The Authors

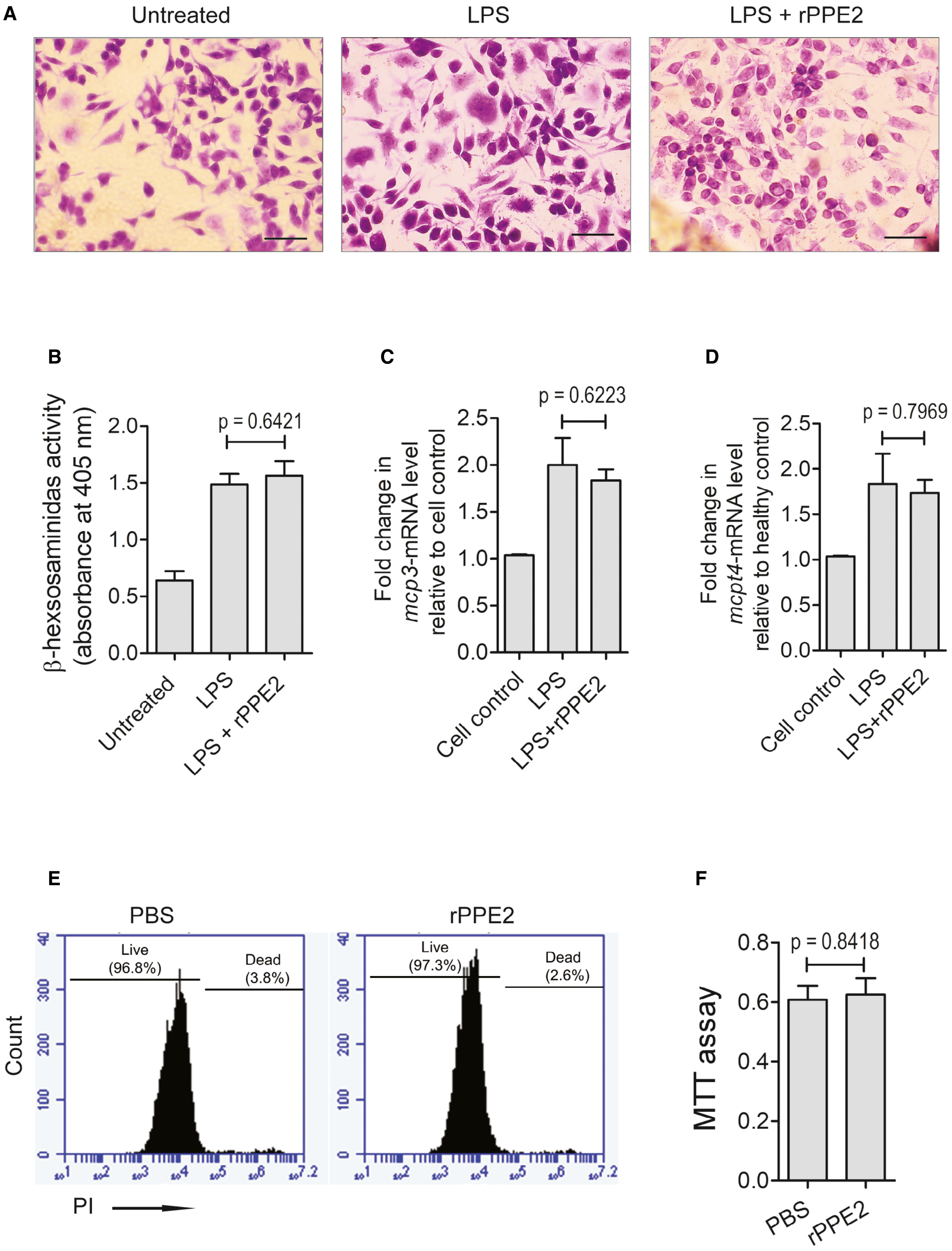

Figure EV3.

 © 2022 The Authors

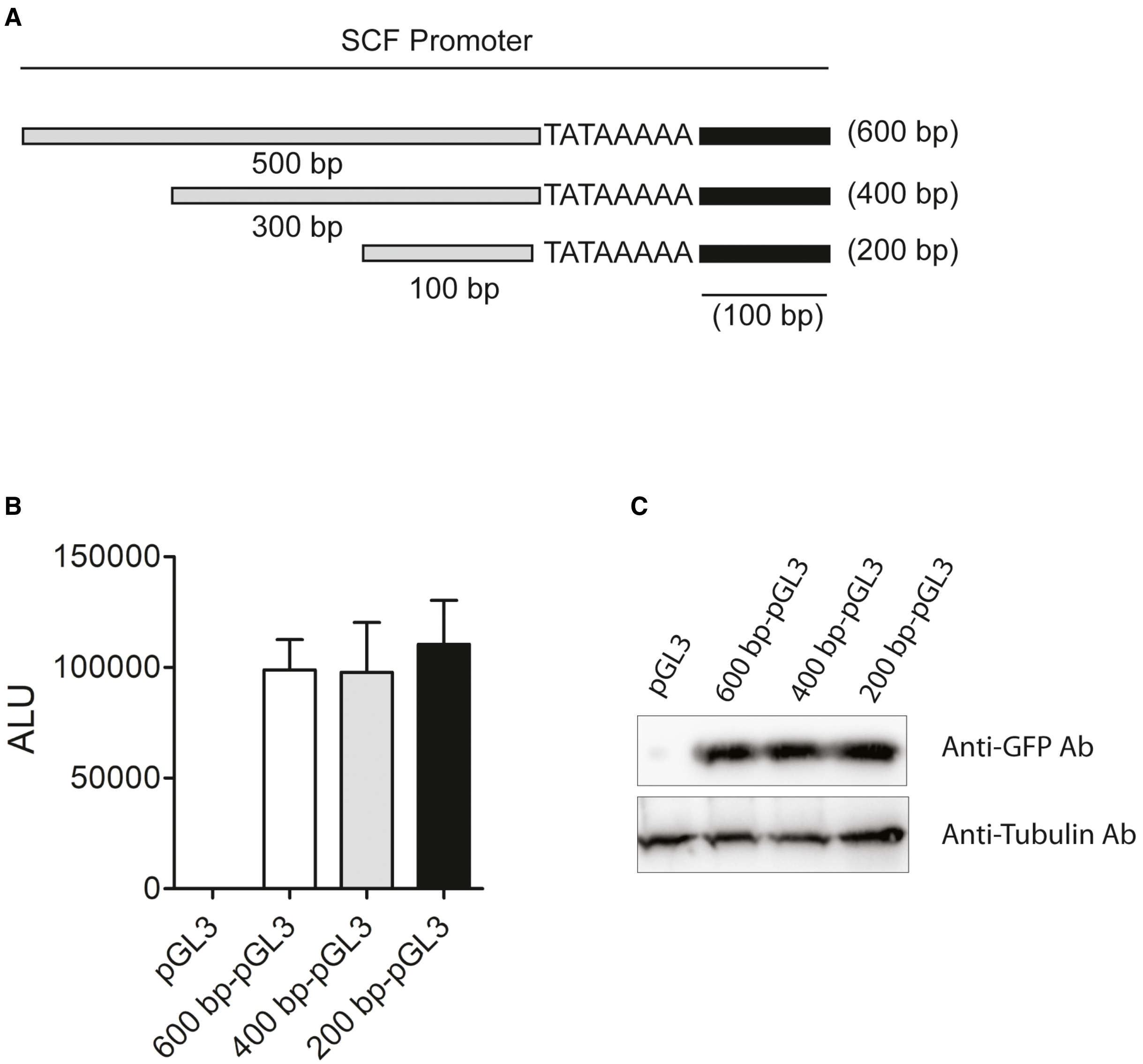

Figure EV4. Mapping of *scf* promoter including transcription start site (TSS).

A Eukaryotic promoter Database (EPD) was used to predict the promoter region of the SCF gene. Three different putative promoter regions, 600 bp (−500 bp, +100 bp), 400 bp (−300 bp, +100 bp) and 200 bp (−100 bp, +100 bp) ranging over the TATAAAA (TSS) of *scf* promoter were selected for analyzing promoter activity.

B These three putative promoters were cloned in pGL3 (promoter-less vector) and transfected in NIH-3T3 cells along with pEGFP. After 24 h of transfection, cells were harvested and analyzed for luciferase activity. Data shown are Mean ± SEM of three independent experiments.

C Lysates prepared from the same samples used in experiment EV5B were resolved on SDS–PAGE and checked for GFP expression by Western blotting using anti-GFP Ab. GAPDH was used as a loading control.

Figure EV5. Designing of PPE2 peptide.

A PPE2 protein of *Mycobacterium tuberculosis* possesses putative leucine zipper (DNA-binding domain) and NLS (highlighted in bold).

B A synthetic peptide of 36 amino acids is synthesized by combining leucine zipper and NLS of PPE2 (KTLLEQTLALLPAALPLLAAPLAPLTLRRRRPKIKQ; Bhat *et al*, 2017).

C Peptide was modeled using MODELLER 9.23.

D The predicted model was verified by Ramachandran plot.

E Peptide was docked with the EPD predicted 60 bp *scf* promoter using online HDOCK tool.

© 2022 The Authors

A

>Mycobacterium tuberculosis H37Rv|Rv0256c|PPE2

MTAPIWMASPPEVHSALLSSGPGPGPLLVSAEGWHSLSIAYAETADELAAL-
LAAVQAGTWDGPTAAVYVAAHTPYLAWLVQASANSAAMATRQETAATAYGTALAAMPTLAELGANHALHGVLMATN
FFGINTIPIALNESDYARMWIQAATTMASYQAVSTAAVAAAPQTTPAPQIVKANAPTAASDEPNQVQEWLQWLQKIGY
TDFYNNVIQPFINWLTNLPFLQAMFSGFDPWLPSLGNPLTFLSPANIAFALGYPMDIGSYVAFLSQTFAFIGADLAAAF
ASGNPATIAFTLMFTTVEAIGTIITDTIALVKT***LLEQTLALLPAALPLLAAPLAPLTLAP***ASAAGGFAGLSGLAGLVGIPP
SAPPVIPPVAAIAPSIPTPTPTPAPAPAPTAVTAPTPPPGPPPPPVTAPPPVTGAGIQSFGYLVGDLNSAAQARKAVGT
GVRKKTPEPDSAEAPAAAAAPEEQVQP***QRRRRPKIK***QLGRGYEYLDLDPETGHDPTGSPQGAGTLGFAGTTHKAS
PGQVAGLITLPNDAFGGSPRTPMMPGTWDTDSATRVE

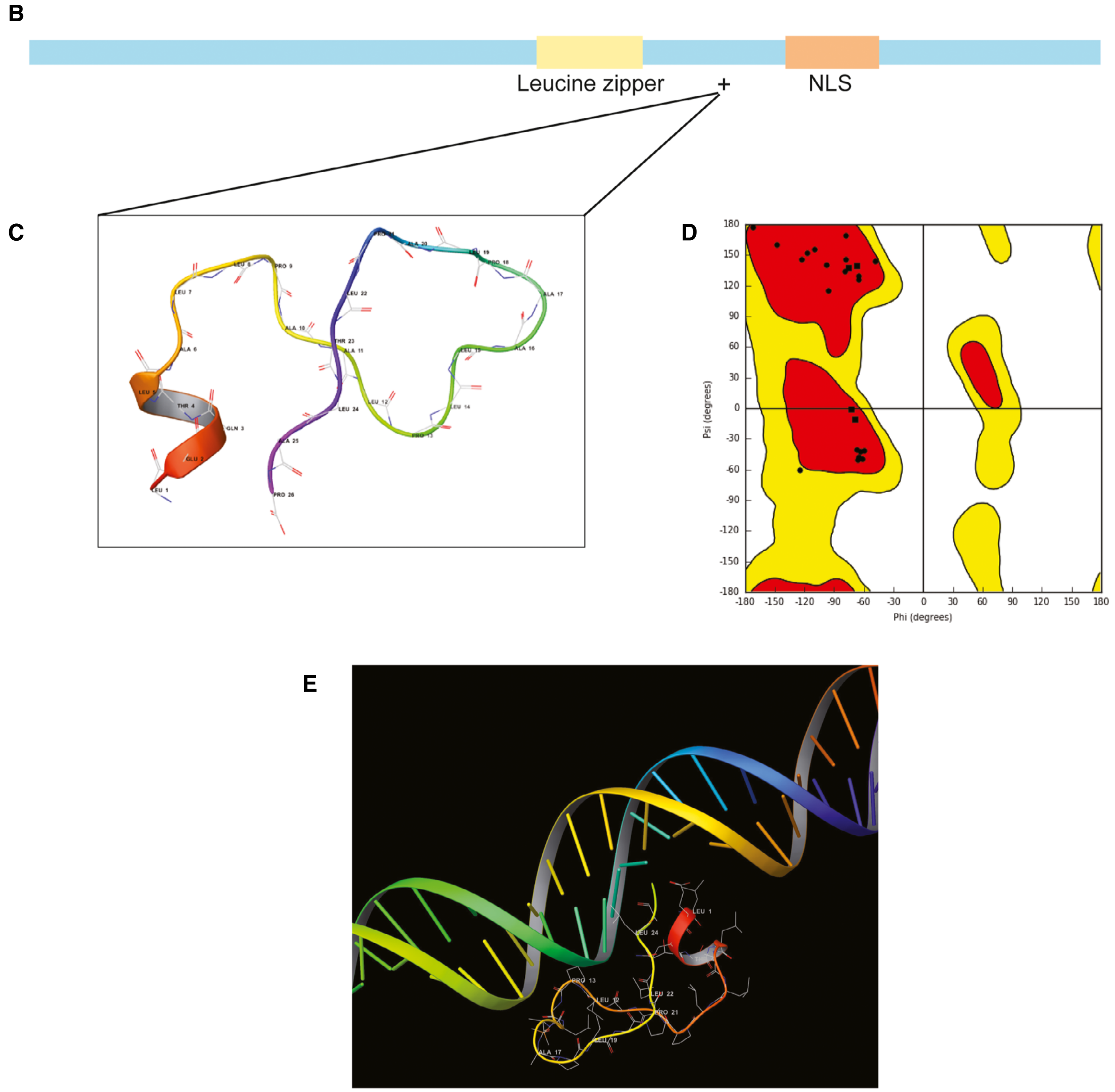

Figure EV5.

© 2022 The Authors
