# Supplementary material for: Therapeutic application of PPE2 protein of Mycobacterium tuberculosis in inhibiting tissue inflammation
Source: EMBO Mol Med. 2022 Jul 11;14(9):e14891. doi: 10.15252/emmm.202114891 (PMC9449591; doi:10.15252/emmm.202114891)
Supplement: Supplementary file 3 [file EMMM-14-e14891-s003.pdf]

EMBO Molecular Medicine

Therapeutic application of PPE2 protein of Mycobacterium tuberculosis in inhibiting tissue inflammation

Ravi Pal, Madhu Battu, and Sangita Mukhopadhyay
DOI: 10.15252/emmm.202114891

Corresponding author: Sangita Mukhopadhyay (sangita@cdfd.org.in)

Review Timeline:

Submission Date:	22nd Jul 21
Editorial Decision:	19th Aug 21
Revision Received:	31st Mar 22
Editorial Decision:	25th May 22
Revision Received:	20th Jun 22
Accepted:	23rd Jun 22

Editor: Zeljko Durdevic

Transaction Report:

(Note: With the exception of the correction of typographical or spelling errors that could be a source of ambiguity, letters and reports are not edited. Depending on transfer agreements, referee reports obtained elsewhere may or may not be included in this compilation. Referee reports are anonymous unless the Referee chooses to sign their reports.)

19th Aug 2021

Dear Dr. Mukhopadhyay,

Thank you for the submission of your manuscript to EMBO Molecular Medicine. We have now received feedback from the two reviewers who agreed to evaluate your manuscript. As you will see from the reports below, the referees acknowledge the interest of the study but also raise important critique that should be addressed in a major revision.

Further consideration of a revision that addresses reviewers' concerns in full will entail a second round of review. EMBO Molecular Medicine encourages a single round of revision only and therefore, acceptance or rejection of the manuscript will depend on the completeness of your responses included in the next, final version of the manuscript. For this reason, and to save you from any frustrations in the end, I would strongly advise against returning an incomplete revision.

We would welcome the submission of a revised version within three to six months for further consideration. However, we realize that the current situation is exceptional on the account of the COVID-19/SARS-CoV-2 pandemic. Please let us know if you require longer to complete the revision.

I look forward to seeing a revised form of your manuscript as soon as possible. Use this link to login to the manuscript system and submit your revision: https://embomolmed.msubmit.net/cgi-bin/main.plex

Yours sincerely,

Zeljko Durdevic

Zeljko Durdevic
Editor
EMBO Molecular Medicine

***** Reviewer's comments *****

Referee #1 (Comments on Novelty/Model System for Author):

Authors should also use Mast cell knockout mice model to address several key questions. Mentioned in the comments to the authors

Referee #1 (Remarks for Author):

In the recent study entitled "Therapeutic application of PPE2 protein of Mycobacterium tuberculosis in inhibiting tissue inflammation" by Pal et al., authors have discovered that PPE2 protein from Mycobacterium tuberculosis (Mtb) suppresses inflammation at the site of injury by inhibiting migration of the mast cell population at injury site. Using formalin -and carrageenan induced paw inflammation model authors showed that PPE2 also inhibits vasoactive fibrogenic and pro-inflammatory cytokines induced by mast cells. Further they showed that PPE2 suppresses inflammation by inhibiting transcription of stem cell factor that is required for mast cell maintenance and migration. Most importantly they designed a synthetic peptide derived from PPE2 and showed that this peptide has anti-inflammatory properties and thus have potential to be used as non-steroidal therapeutic protein for treatment of inflammation and and tissue injury. Though the study is of significant importance, manuscript is poorly written with many grammatical errors, missing links between the paragraphs. There are some major concerns which need to be addressed.

Major Concerns:

1. It has been reported before by authors that PPE18 and peptide derived from PPE18 suppresses sepsis induced inflammation. Authors should determine if PPE18 or PPE2 will have similar effects in suppressing formalin or carrageenan induced paw inflammation model or this phenotype is specific to PPE2.
2. Authors have mentioned that they have seen PPE2 localization in hypodermal and dermal region therefore they have used NIH-3T3 cell line for localization studies. Data and results can vary from cell line to cell line and its essential that authors should perform studies mentioned in Fig. 4 (B-E) in another fibroblast cell line as well.
3. In all the studies performed either the recombinant protein PPE2 or peptide derived from PPE2 was given after 1 hour of formalin induced injury. Authors have not demonstrated or discussed if PPE2 or peptide derived from PPE2 will have therapeutic effects in suppressing the inflammation if treatment is given after 3 hour or 24 hour after the injury.
4. Authors have always relied on the RT-PCR to determine that inhibition of migration of mast cells to the injury site leads to decrease in proinflammatiry cytokine load in the tissue. Authors should also use tissue lysate to determine that the decrease in proinflammatory cytokines at protein level. Also they should determine if the decrease in levels of these cytokines is limited to paw tissue or they also observe decrease in the total serum.
5. It has been mentioned in line 347-348 that there is mast cell knock out mice model. Authors should use that model to demonstrate that PPE2 suppresses formalin induced tissue inflammation specifically through mast cells. As that is the major finding of the manuscript.
6. Discussion needs to be significantly modified and rewritten .
7. In Fig.1C and 2C, only representative pathology pictures are provided. Authors should provide as a graph overall pathology scores per paw. That will provide overall representation of the outcomes of the treatments.
8. Hematoxylin and eosin stained sections in treatment group rPPE2 at 3 hours (Fig.1 C) or 21 hours (Fig. 2C) are similar in representative images presented for pathology. However in Fig. 2A there is major difference in paw inflammation at 3 vs 21 hours. Authors have not provided any explanation for this.
9. In Fig. 3 (A and B) authors have showed representative pictures of stained tissue to show that PPE2 depletes mast cell population in the inflamed paw tissue . It is essential to show quantitatively using FACS, that they see similar decrease in mast cell population in paw.
10. No explanation is provided why authors have not used untreated control in Fig. 3 (E and F).

Minor concerns:
1. Line no: 65; Repeated word "in" and sentence need to be rewritten.
2. Line 146: "Total" should be replaced with "total".

3. Line 171: "Once activated," should be changed to Activated mast cells...
4. Line 181: "reveal" should be changed to "revealed"
5. Authors should explain what they mean by small molecules in Line 417 as they mentioned peptide also as a small molecule (line 414), sentence is contradictory.
6. Line 431: "Thus PPE2/peptide" should be "Thus, PPE2/peptide"

Referee #2 (Remarks for Author):

Work by Pal et al demonstrates the role of synthetic/recombinant mycobacterial protein rPPE2 in ameliorating inflammation in murine model in the context of formalin/carrageenan treatment. The authors hereby made an attempt to decipher the mechanistic basis of rPPE2 action. rPPE2 has been demonstrated to reduce the abundance of mast cells in the tissue undergoing inflammation-induced tissue damage. Mast cell-associated mediators like beta-hexosaminidase activity, MCP-3 and 4 levels in formalin-treated tissue were shown to be significantly downregulated in the presence of rPPE2 treatment. Moreover, the release of mast cell granule contents was observed to be reduced upon treatment with the recombinant mycobacterial peptide. Using in vitro system, the authors showed that the activity of rPPE2 was dependent upon the action of the peptide upon fibroblast rather than mast cells per se. rPPE2 reduced SCF transcription in fibroblasts by inhibiting SCF promoter activity thus reducing mast cell recruitment to the site of injury and mast cell survival. However, prior to acceptance, few minor questions needed to be addressed are as follows:
(1) Authors administered recPPE2 post 1 hour of formalin treatment which resulted in reduction of magnitude of inflammation-associated tissue damage. It would be interesting to see the range of time period following formalin-treatment during which administration of recPPE2 successfully abrogated inflammation-induced tissue damage.

(2) The authors checked the effect of recPPE2 and diclofenac upon liver and kidney to compare their toxicity. In the manuscript, the authors have been observed to use diclofenac at a dose of 10mg/kg of body-weight which demonstrate toxicity at the aforementioned dosage. Was diclofenac dosage below 10mg/kg bodyweight incapable of reducing paw inflammation at 21 days post formalin treatment as shown in fig: 2A? If diclofenac at low dosage is successful in reducing long term inflammation post formalin treatment, comparison of the toxicity of low dosage diclofenac treatment with that of rec PPE2-treatment would be more accurate.

(3) Authors in fig: S5 B transfected cells with pGL3-600bp/400bp/200bp and checked as well as compared their luciferase activity. However, since pGL3 vector contains a single luciferase enzyme, normalization of the Pgl3-derived luciferase activity using another reporter activity (by co-transfecting pGL3-transfecting cells with another plasmid expressing renilla luciferase activity/EGFP) would help to rule out unequal transfection efficiency of pGL3 vector containing 600/400/200bp inserts.

(4) The authors should revise and correct multiple grammatical mistakes in manuscript that would make the scientific-story presented by the manuscript more appealing to the readers.

Point by point response

Referee #1 (Remarks for Author):

In the recent study entitled "Therapeutic application of PPE2 protein of *Mycobacterium tuberculosis* in inhibiting tissue inflammation" by Pal et al., authors have discovered that PPE2 protein from *Mycobacterium tuberculosis* (Mtb) suppresses inflammation at the site of injury by inhibiting migration of the mast cell population at injury site. Using formalin -and carrageenan-induced paw inflammation model authors showed that PPE2 also inhibits vasoactive fibrogenic and pro-inflammatory cytokines induced by mast cells. Further, they showed that PPE2 suppresses inflammation by inhibiting transcription of stem cell factor that is required for mast cell maintenance and migration. Most importantly they designed a synthetic peptide derived from PPE2 and showed that this peptide has anti-inflammatory properties and thus have potential to be used as non-steroidal therapeutic protein for treatment of inflammation and tissue injury. Though the study is of significant importance, manuscript is poorly written with many grammatical errors, missing links between the paragraphs. There are some major concerns which need to be addressed.

Major Concerns:

Query 1. *It has been reported before by authors that PPE18 and peptide derived from PPE18 suppresses sepsis induced inflammation. Authors should determine if PPE18 or PPE2 will have similar effects in suppressing formalin or carrageenan induced paw inflammation model or this phenotype is specific to PPE2.*

Our response. We thank the esteemed referee for this interesting suggestion as both PPE18 and PPE2 proteins are anti-inflammatory in nature, it would be interesting to compare their activities in formalin-induced inflammation model. Earlier we demonstrated that recombinantly purified PPE18 (rPPE18) protein reduces sepsis-induced mortality (Ahmed *et al*., 2018, Journal of Immunology, 200: 3587-3598) and it binds to TLR2 to inhibit LPS-induced production of TNF-α and IL-12 with simultaneous upregulation of IL-10 (Nair *et al*., 2009, Journal of Immunology 183: 6269-6281 and Nair *et al*., 2011, Journal of Immunology 186: 5413-5424). As suggested by the referee, in the revised manuscript, we compared the effect of rPPE18 and rPPE2 (recombinantly purified PPE2) to suppress formalin-induced paw inflammation. To test this, a subplantar injection of 5% of formalin (20 µl) was given in the right hind paw of Balb/c mice, and an equal volume of PBS was injected in left hind paw. After 1 hour of formalin injection, mice were administered intraperitoneally with a single dose of either rPPE2 (3 mg/kg) or rPPE18 (3 mg/kg) or PBS (vehicle control) and paw inflammation was measured after 3 hours of rPPE2/rPPE18 treatment. We observed that more than 50% reduction in inflammation by rPPE2 as compared to only about 20% by rPPE18 and rPPE18 significantly failed to reduce the mast cell population in the inflamed tissue. These results clearly indicate that rPPE18 is less efficient in suppressing formalin-induced inflammation (please see Appendix Figure S2 and page no 10, line no 185-194; page 21-22, line no 428-434 of the revised manuscript).

Query 2. *Authors have mentioned that they have seen PPE2 localization in hypodermal and dermal region therefore they have used NIH-3T3 cell line for localization studies. Data and results can vary from cell line to cell line and it's essential that authors should perform studies mentioned in Fig. 4 (B-E) in another fibroblast cell line as well.*

Our response. We again thank the esteemed referee for this suggestion. Accordingly, we have performed confocal microscopy to investigate nuclear localization of rPPE2 using another fibroblast cells line, mouse embryo fibroblasts (MEFs). MEFs were maintained and cultured in DMEM (supplemented with 10% FBS, 1X Gultamax and 1X Anti-Anti). To observe localization of PPE2, MEFs were incubated with 3 µg/ml rPPE2 for 45 minutes, cells were permeabilized using 0.1% Triton X-100 and incubated with anti-PPE2 Ab [in-house raised, (Bhat *et al*., 2017)] followed by incubation with Alexa Fluor 488–conjugated anti-mouse secondary Ab. Cells were washed and mounted with a mounting medium containing DAPI (VECTASHIELD) and observed under LSM 700 Zeiss confocal microscope. We observed that PPE2 was translocated to the nucleus of another fibroblast cells line (MEF) also. (Kindly refer to Figure 5D, E; page no 13, line no 255-257; page no 29, line no 590 and page no 47, lines 971-972 of the revised manuscript).

Query 3. *In all the studies performed either the recombinant protein PPE2 or peptide derived from PPE2 was given after 1 hour of formalin induced injury. Authors have not demonstrated or discussed if PPE2 or peptide derived from PPE2 will have therapeutic effects in suppressing the inflammation if treatment is given after 3 hour or 24 hour after the injury.*

Our response. As suggested by the referee, we have checked the efficacy of rPPE2 to suppress the formalin-induced paw inflammation when administered after 3 hours or 24 hours or 48 hours of injury. Hind paw of Balb/c mice of 6-8 weeks of age was injected with 5% formalin. After 3 hours or 24 hours or 48 hours, mice were administered intraperitoneally with a single dose of either rPPE2 (3 mg/kg) or PBS (vehicle control) and inflammation was recorded after 3 hours of treatment. We observed that mice had a reduction in paw

inflammation even when rPPE2 was administered after 3 hours as well as after 24 and 48 hours of formalin-induced tissue injury. This suggests the ability of rPPE2 to act on an established full-fledged inflammation (Kindly refer to Figure 1K; page no 6, line no 106-108; page 20, lines 400-402 and page no 44, lines 905-909 of the revised manuscript)

Query 4. *Authors have always relied on the RT-PCR to determine that inhibition of migration of mast cells to the injury site leads to decrease in pro-inflammatory cytokine load in the tissue. Authors should also use tissue lysate to determine that the decrease in pro-inflammatory cytokines at protein level. Also they should determine if the decrease in levels of these cytokines is limited to paw tissue or they also observe decrease in the total serum.*

Our response. We thank the esteemed referee for this suggestion. The levels of TNF-α and IL-6 were checked in paw tissue lysates and whole blood sera by EIA. Briefly, the paw tissues were harvested from various groups and tissue lysates were prepared. An equal amount of lysates (500 µg) from each group were used to determine the TNF-α and IL-6 cytokines at protein levels by sandwich EIA (Invitrogen, USA). Also, the serum levels of TNF-α and IL-6 cytokines were checked by EIA. As expected, we observed a reduction in the levels of TNF-α and IL-6 in tissue lysates as well as in sera of mice treated with rPPE2 (3 mg/kg) when compared with PBS control (vehicle control) group (Kindly refer to Figure 1H and 1I and Appendix Figure S1; page no 6, line no 96-97 and 98-99; page 20, line no 398; page no 29-30, line no 601-605 and page no 43, lines 901-903 of the revised manuscript)

Query 5. *It has been mentioned in line 347-348 that there is mast cell knock out mice model. Authors should use that model to demonstrate that PPE2 suppresses formalin induced tissue inflammation specifically through mast cells. As that is the major finding of the manuscript.*

Our response. We appreciate this suggestion by the referee. We have mentioned in our discussion that Lee *et al*., (2002) have described that mast cell knockout mice failed to develop joint inflammation/arthritis, but the susceptibility was restored when mice were engrafted with the mast cells (Lee *et al*., 2002). In this study, we demonstrated that PPE2 inhibits mast cells and as mast cells are important to mount an effective inflammatory response, PPE2 may be useful as a non-steroidal anti-inflammatory drug. In this context, studies using mast cell knock-out mice are appreciated. However, due to COVID-19 pandemic regulations, we deeply regret our inability to procure Mast cell knock-out mice to carry out the suggested experiment within the required time. However, to confirm our findings that rPPE2 predominantly targets the mast cells to reduce paw inflammation and tissue injury, we have carried out the transplantation of mast cells experiment as described elsewhere (Lee *et al*., 2002; Patel *et al*., 2016; Habuchi *et al*., 2021). The bone marrow-derived mast cells were cultured as described elsewhere (Meurer *et al*., 2016), and about 1×10^6 viable bone marrow-derived mast cells (> 95% purity) were injected *via* the subplantar route in the right hind paw of mice treated with rPPE2 (3 mg/kg). We observed that after 3 hours of mast cells transfer, the anti-inflammatory effect of PPE2 was reversed and the paw tissues regained redness and swelling/inflammation. These results support the fact that PPE2 suppresses formalin-induced tissue inflammation specifically by reducing mast cells population in the inflamed paw tissue (please see Figure 4 and page no 11-12, line no 207-223; page no 21, line no 424-426; page 28, lines 579-587; page 30, lines 614-621 and page no 46, lines 950-962 of the revised manuscript.

Query 6. *Discussion needs to be significantly modified and rewritten.*

Our response. As suggested discussion is modified and rewritten in the revised manuscript and highlighted for the referee's perusal (please see page no 18-25, line no 367-501 of the revised manuscript.

Query 7. *In Fig.1C and 2C, only representative pathology pictures are provided. Authors should provide as a graph overall pathology scores per paw. That will provide overall representation of the outcomes of the treatments.*

Our response. As suggested we have now incorporated a graph that represents overall pathology scores per paw (please see Figure 1E and page no 5-6, line no 90-93; page no 28 line no 562-565 and page no 43, lines 895-898 of the revised manuscript)

Query 8. *Hematoxylin and eosin stained sections in treatment group rPPE2 at 3 hours (Fig.1 C) or 21 hours (Fig. 2C) are similar in representative images presented for pathology. However in Fig. 2A there is major difference in paw inflammation at 3 vs 21 hours. Authors have not provided any explanation for this.*

Our response. We are sorry for this inadvertent mistake. We have carried out new experiments and incorporated new pictures (please see Figure 2C of the revised manuscript)

Query 9. *In Fig. 3 (A and B) authors have showed representative pictures of stained tissue to show that PPE2 depletes mast cell population in the inflamed paw tissue. It is essential to show quantitatively using FACS, that they see similar decrease in mast cell population in paw.*

Our response. We thank the esteemed referee for this valuable suggestion. As suggested, in the revised manuscript flow cytometry analysis was performed to determine the mast cells population in the paw tissues of various groups. CD117 and FcεRI were used as the markers of the tissue-resident mast cells. As expected, mice treated with 3 mg/kg of rPPE2 had reduction in mast cell population in paw tissue as compared to the vehicle control which supports our earlier findings based on Toluidine blue stained tissue sections (please see Figure 3C, D and page no 9-10, line no 177-181 and page no 28, line no 567-577 and page no 45, lines 935-941 of the revised manuscript).

Query 10. *No explanation is provided why authors have not used untreated control in Fig. 3 (E and F).*

Our response. The comparative Ct method ($2^{-\Delta\Delta}$Ct) was used to calculate the relative change in mRNA levels using expression from healthy control as base (1 fold) as described elsewhere (Livak & Schmittgen, 2001). Briefly, ΔCt is the Ct value for the gene of interest normalized to the Ct value of the respective GAPDH control. ΔΔCt values were calculated as a relative change in ΔCt of the target gene in all the groups with respect to healthy control. Fold changes in the mRNA levels were expressed as $2^{-\Delta\Delta Ct}$.

As suggested by the esteemed reviewer, we have now included a bar representing the fold change values of healthy controls over itself. To better illustrate this calculation, we re-labeled the y-axis of graphs shown in Figures 3E and 3F as "Fold change in 'MCP-3/Mcpt4' mRNA levels relative to healthy control". The detailed description is now included in the 'Materials and Methods section' of the revised manuscript (please see page no 31-32, lines 641-647 of the revised manuscript).

Minor concerns:

Query 1. Line no: 65; Repeated word "in" and sentence need to be rewritten.

Our response. This sentence is deleted in the revised manuscript.

Query 2. Line 146: "Total" should be replaced with "total".

Our response. We have corrected this in the revised manuscript (please see page no 8, line no 156).

Query 3. Line 171: "Once activated," should be changed to Activated mast cells...

Our response. We have corrected this in the revised manuscript (please see page no 10, line no 196)

Query 4. Line 181: "reveal" should be changed to "revealed"

Our response. We have rewritten this line in the revised manuscript (please see page no 11, line no 207-208).

Query 5. Authors should explain what they mean by small molecules in Line 417 as they mentioned peptide also as a small molecule (line 414), sentence is contradictory.

Our response. We are sorry for this inadvertent mistake. The discussion chapter is re-written and we have taken care of this mistake.

Query 6. Line 431: "Thus PPE2/peptide" should be "Thus, PPE2/peptide"

Our response. We have incorporated this correction in the revised manuscript (please see page no 25, line no 499 of the revised manuscript).

Referee #2 (Remarks for Author):

Work by Pal et al demonstrates the role of synthetic/recombinant mycobacterial protein rPPE2 in ameliorating inflammation in murine model in the context of formalin/carrageenan treatment. The authors hereby made an attempt to decipher the mechanistic basis of rPPE2 action. rPPE2 has been demonstrated to reduce the abundance of mast cells in the tissue undergoing inflammation-induced tissue damage. Mast cell-associated mediators like beta-hexosaminidase activity, MCP-3 and 4 levels in formalin-treated tissue were shown to be significantly downregulated in the presence of rPPE2 treatment. Moreover, the release of mast cell granule contents was observed to be reduced upon treatment with the recombinant mycobacterial peptide. Using in vitro system, the authors showed that the activity of rPPE2 was dependent upon the action of the peptide upon fibroblast rather than mast cells per se. rPPE2 reduced SCF transcription in fibroblasts by inhibiting SCF promoter activity thus reducing mast cell recruitment to the site of injury and mast cell survival. However, prior to acceptance, few minor questions needed to be addressed are as follows:

Query 1. *Authors administered recPPE2 post 1 hour of formalin treatment which resulted in reduction of magnitude of inflammation-associated tissue damage. It would be interesting to see the range of time period following formalin-treatment during which administration of recPPE2 successfully abrogated inflammation-induced tissue damage.*

Our response. This query is also raised by Referee 1. As suggested by both the referees, we have checked the efficacy of rPPE2 to suppress the formalin-induced paw inflammation when administered after 3 hours or 24 hours or 48 hours post-injury. Right hind paw of Balb/c mice of 6-8 weeks of age was injected with 5% formalin. After 3 hours or 24 hours or 48 hours, mice were administered intraperitoneally with a single dose of either rPPE2 (3 mg/kg) or PBS (vehicle control). and inflammation was recorded. We observed that mice had a reduction in

paw inflammation even when rPPE2 was administered after 3 hours as well as after 24 and 48 hours of formalin-induced tissue injury. This suggests the ability of rPPE2 to act on an established full-fledged inflammation (Kindly refer to Figure 1K; page no 6, line no 106-108; page 20, lines 400-402 and page no 44, lines 905-909 of the revised manuscript)

Query 2. *The authors checked the effect of recPPE2 and diclofenac upon liver and kidney to compare their toxicity. In the manuscript, the authors have been observed to use diclofenac at a dose of 10mg/kg of body-weight which demonstrate toxicity at the aforementioned dosage. Was diclofenac dosage below 10mg/kg bodyweight incapable of reducing paw inflammation at 21 days post formalin treatment as shown in fig: 2A? If diclofenac at low dosage is successful in reducing long term inflammation post formalin treatment, comparison of the toxicity of low dosage diclofenac treatment with that of rec PPE2-treatment would be more accurate.*

Our response. We thank the esteemed reviewer for this suggestion. Administration of Diclofenac at 10 mg/kg is a standard and widely used drug concentration [Yin *et al.*, 2016]. Therefore, we used 10 mg/kg of Diclofenac as a standard drug control in all experiments. However, as suggested by the reviewer, in the revised manuscript, we have compared the efficacy of Diclofenac to reduce long-term paw tissue inflammation at both lower dose (3 mg/kg) and higher dose (10 mg/kg) in Figure 2 of the revised manuscript. We observed that mice administered with 10 mg/kg **BUT NOT** 3 mg/kg dose of Diclofenac showed an almost similar reduction of long-term inflammation as that of 3 mg/kg of rPPE2 (please see Figure 2 and page no 7, line no 130; page no 7-8, lines 134-143; page no 8, lines 149-152 and page no 44, lines 912-924 of the revised manuscript). Thus, a comparison of the toxicity of 10 mg/kg

dosage diclofenac treatment with that of 3 mg/kg of rPPE2 treatment in Figure EV2 is justified (page no 8, line no 149-152).

Query 3. *Authors in fig: S5 B transfected cells with pGL3-600bp/400bp/200bp and checked as well as compared their luciferase activity. However, since pGL3 vector contains a single luciferase enzyme, normalization of the Pgl3-derived luciferase activity using another reporter activity (by co-transfecting pGL3-transfecting cells with another plasmid expressing renilla luciferase activity/EGFP) would help to rule out unequal transfection efficiency of pGL3 vector containing 600/400/200bp inserts.*

Our response. We thank the reviewer for this valuable input. As suggested, pEGFP plasmid was used as transfection control to rule out unequal transfection efficiency of pGL3 vector containing 600/400/200bp inserts (please see Figure EV4C and page no 15, line no 292-294 of the revised manuscript.

Query 4. *The authors should revise and correct multiple grammatical mistakes in manuscript that would make the scientific-story presented by the manuscript more appealing to the readers.*

Our response. As suggested, the grammatical errors have been amended in the revised manuscript.

List of references

Ahmed A, Dolasia K, Mukhopadhyay S (2018) *Mycobacterium tuberculosis* PPE18 protein reduces inflammation and increases survival in animal model of sepsis. Journal of immunology (Baltimore, Md : 1950) 200: 3587-3598

Bhat KH, Srivastava S, Kotturu SK, Ghosh S, Mukhopadhyay S (2017) The PPE2 protein of *Mycobacterium tuberculosis* translocates to host nucleus and inhibits nitric oxide production. *Scientific reports* 7: 39706

Habuchi H, Izumi M, Dan J, Ushida T, Ikeuchi M, Takeuchi K, Habuchi O (2021) Bone marrow derived mast cells injected into the osteoarthritic knee joints of mice induced by sodium monoiodoacetate enhanced spontaneous pain through activation of PAR2 and action of extracellular ATP. PloS one 16: e0252590

Lee DM, Friend DS, Gurish MF, Benoist C, Mathis D, Brenner MB (2002) Mast cells: a cellular link between autoantibodies and inflammatory arthritis. *Science (New York, NY)* 297: 1689-1692

Livak KJ, Schmittgen TD (2001) Analysis of relative gene expression data using real-time quantitative PCR and the 2(-Delta Delta C(T)) Method. *Methods (San Diego, Calif)* 25: 402-408

Meurer SK, Neß M, Weiskirchen S, Kim P, Tag CG, Kauffmann M, Huber M, Weiskirchen R (2016) Isolation of mature (peritoneum-derived) mast cells and immature (Bone Marrow-Derived) mast cell precursors from mice. *PloS one* 11: e0158104

Nair S, Pandey AD, Mukhopadhyay S (2011) The PPE18 protein of Mycobacterium tuberculosis inhibits NF-κB/rel-mediated proinflammatory cytokine production by upregulating and phosphorylating suppressor of cytokine signaling 3 protein. Journal of immunology (Baltimore, Md : 1950) 186: 5413-5424

Nair S, Ramaswamy PA, Ghosh S, Joshi DC, Pathak N, Siddiqui I, Sharma P, Hasnain SE, Mande SC, Mukhopadhyay S (2009) The PPE18 of *Mycobacterium tuberculosis* interacts with TLR2 and activates IL-10 induction in macrophage. Journal of immunology (Baltimore, Md : 1950) 183: 6269-6281

Patel KR, Aven L, Shao F, Krishnamoorthy N, Duvall MG, Levy BD, Ai X (2016) Mast cell-derived neurotrophin 4 mediates allergen-induced airway hyperinnervation in early life. *Mucosal immunology* 9: 1466-1476

Yin ZY, Li L, Chu SS, Sun Q, Ma ZL, Gu XP (2016) Antinociceptive effects of dehydrocorydaline in mouse models of inflammatory pain involve the opioid receptor and inflammatory cytokines. Scientific reports 6: 27129

25th May 2022

Dear Dr. Mukhopadhyay,

Thank you for the submission of your revised manuscript to EMBO Molecular Medicine, and please accept my apologies for the delay in getting back to you. I am pleased to inform you that we will be able to accept your manuscript pending the following final amendments:

1) Figures: Please submit all EV Figures as separate, high-resolution files and add their legends at the end of the main manuscript file.
2) In the main manuscript file, please do the following:
- Correct/answer the track changes suggested by our data editors by working from the attached document.
- Limit keywords to max 5.
- Remove text highlight colour.
- Make sure that all special characters display well.
- Add callouts for Figure 4H.
- Add "The Paper Explained" to the main manuscript file.
- In M&M, provide the antibody dilutions that were used for each antibody.
- In M&M, a statistical paragraph that should reflect all information that you have filled in the Authors Checklist, especially regarding randomization, blinding, replication.
3) Appendix: Please add Table of Content.
4) Conflict of interest: We updated our journal's competing interests policy in January 2022 and request authors to consider both actual and perceived competing interests. Please review the policy https://www.embopress.org/competing-interests and update your competing interests if necessary
5) Synopsis: Every published paper now includes a 'Synopsis' to further enhance discoverability. Synopses are displayed on the journal webpage and are freely accessible to all readers. They include separate synopsis image and synopsis text.
- Synopsis text: Please provide a short stand first (maximum of 300 characters, including space) as well as 2-5 one sentence bullet points that summarise the paper as a .doc file. Please write the bullet points to summarise the key NEW findings. They should be designed to be complementary to the abstract - i.e. not repeat the same text. We encourage inclusion of key acronyms and quantitative information (maximum of 30 words / bullet point). Please use the passive voice.
- Synopsis image: Please provide a striking image or visual abstract as a high-resolution jpeg file 550 px-wide x (250-400)-px high to illustrate your article.
- Please check your synopsis text and image and submit their final versions with your revised manuscript. Please be aware that in the proof stage minor corrections only are allowed (e.g., typos).
6) For more information: There is space at the end of each article to list relevant web links for further consultation by our readers. Could you identify some relevant ones and provide such information as well? Some examples are patient associations, relevant databases, OMIM/proteins/genes links, author's websites, etc...
7) Source data: We encourage you to include the source data for figure panels that show essential data. Numerical data should be provided as individual .xls or .csv files (including a tab describing the data). For blots or microscopy, uncropped images should be submitted (using a zip archive if multiple images need to be supplied for one panel). Please check "Author Guidelines" for more information. https://www.embopress.org/page/journal/17574684/authorguide#sourcedata
8) As part of the EMBO Publications transparent editorial process initiative (see our Editorial at http://embomolmed.embopress.org/content/2/9/329), EMBO Molecular Medicine will publish online a Review Process File (RPF) to accompany accepted manuscripts. This file will be published in conjunction with your paper and will include the anonymous referee reports, your point-by-point response and all pertinent correspondence relating to the manuscript. Let us know whether you agree with the publication of the RPF and as here, if you want to remove or not any figures from it prior to publication. Please note that the Authors checklist will be published at the end of the RPF.
9) Please provide a point-by-point letter INCLUDING my comments as well as the reviewer's reports and your detailed responses (as Word file).

I look forward to reading a new revised version of your manuscript as soon as possible.

Yours sincerely,

Zeljko Durdevic

Zeljko Durdevic
Editor
EMBO Molecular Medicine

***** Reviewer's comments *****

Referee #1 (Remarks for Author):

As authors have addressed concerns raised by the reviewers this manuscript is acceptable.

Referee #2 (Comments on Novelty/Model System for Author):

The manuscript has been improved significantly from the first submission.

Referee #2 (Remarks for Author):

I am satisfied with revision work undertaken by the authors

The authors performed the requested editorial changes.

2nd Revision - Editorial Decision 23rd Jun 2022

We are pleased to inform you that your manuscript is accepted for publication and is now being sent to our publisher to be included in the next available issue of EMBO Molecular Medicine.

EMBO Press Author Checklist

Corresponding Author Name: Dr. Sangita Mukhopadhyay	
Journal Submitted to: EMBO Molecular Medicine	
Manuscript Number: EMM-2021-14891	

USEFUL LINKS FOR COMPLETING THIS FORM
The EMBO Journal - Author Guidelines
EMBO Reports - Author Guidelines
Molecular Systems Biology - Author Guidelines
EMBO Molecular Medicine - Author Guidelines

Reporting Checklist for Life Science Articles (updated January 2022)
This checklist is adapted from Materials Design Analysis Reporting (MDAR) Checklist for Authors. MDAR establishes a minimum set of requirements in transparent reporting in the life sciences (see Statement of Task: 10.31222/osf.io/9sm4x). Please follow the journal's guidelines in preparing your manuscript.
Please note that a copy of this checklist will be published alongside your article.

Abridged guidelines for figures
1. Data
The data shown in figures should satisfy the following conditions:
- the data were obtained and processed according to the field's best practice and are presented to reflect the results of the experiments in an accurate and unbiased manner.
- ideally, figure panels should include only measurements that are directly comparable to each other and obtained with the same assay.
- plots include clearly labeled error bars for independent experiments and sample sizes. Unless justified, error bars should not be shown for technical replicates.
- if n<5, the individual data points from each experiment should be plotted. Any statistical test employed should be justified.
- Source Data should be included to report the data underlying figures according to the guidelines set out in the authorship guidelines on Data Presentation.

2. Captions
Each figure caption should contain the following information, for each panel where they are relevant:
- a specification of the experimental system investigated (eg cell line, species name).
- the assay(s) and method(s) used to carry out the reported observations and measurements.
- an explicit mention of the biological and chemical entity(ies) that are being measured.
- an explicit mention of the biological and chemical entity(ies) that are altered/varied/perturbed in a controlled manner.
- the exact sample size (n) for each experimental group/condition, given as a number, not a range;
- a description of the sample collection allowing the reader to understand whether the samples represent technical or biological replicates (including how many animals, litters, cultures, etc.).
- a statement of how many times the experiment shown was independently replicated in the laboratory.
- definitions of statistical methods and measures:
  - common tests, such as t-test (please specify whether paired vs. unpaired), simple χ2 tests, Wilcoxon and Mann-Whitney tests, can be unambiguously identified by name only, but more complex techniques should be described in the methods section;
  - are tests one-sided or two-sided?
  - are there adjustments for multiple comparisons?
  - exact statistical test results, e.g., P values = x but not P values < x;
  - definition of 'center values' as median or average;
  - definition of error bars as s.d. or s.e.m.

Please complete ALL of the questions below.
Select "Not Applicable" only when the requested information is not relevant for your study.

Materials

Newly Created Materials	Information included in the manuscript?	In which section is the information available? (Reagents and Tools Table, Materials and Methods, Figures, Data Availability Section)
New materials and reagents need to be available; do any restrictions apply?	Yes	Materials and Methods

Antibodies	Information included in the manuscript?	In which section is the information available? (Reagents and Tools Table, Materials and Methods, Figures, Data Availability Section)
For **antibodies** provide the following information:<br>- Commercial antibodies: RRID (if possible) or supplier name, catalogue number and or/clone number<br>- Non-commercial: RRID or citation	Yes	Materials and Methods

DNA and RNA sequences	Information included in the manuscript?	In which section is the information available? (Reagents and Tools Table, Materials and Methods, Figures, Data Availability Section)
**Short novel DNA or RNA including primers, probes**: provide the sequences.	Yes	Table 1

Cell materials	Information included in the manuscript?	In which section is the information available? (Reagents and Tools Table, Materials and Methods, Figures, Data Availability Section)
**Cell lines:** Provide species information, strain. Provide accession number in repository **OR** supplier name, catalog number, clone number, and/**OR** RRID.	Yes	Materials and Methods
**Primary cultures:** Provide species, strain, sex of origin, genetic modification status.	Yes	Materials and Methods
Report if the cell lines were recently **authenticated** (e.g., by STR profiling) and tested for mycoplasma contamination.	Yes	Materials and Methods

Experimental animals	Information included in the manuscript?	In which section is the information available? (Reagents and Tools Table, Materials and Methods, Figures, Data Availability Section)
**Laboratory animals or Model organisms:** Provide species, strain, sex, age, genetic modification status. Provide accession number in repository **OR** supplier name, catalog number, clone number, **OR** RRID.	Yes	Materials and Methods
**Animal observed in or captured from the field:** Provide species, sex, and age where possible.	Not Applicable	
Please detail **housing and husbandry conditions**.	Yes	Materials and Methods

Plants and microbes	Information included in the manuscript?	In which section is the information available? (Reagents and Tools Table, Materials and Methods, Figures, Data Availability Section)
**Plants:** provide species and strain, ecotype and cultivar where relevant, unique accession number if available, and source (including location for collected wild specimens).	Not Applicable	
**Microbes:** provide species and strain, unique accession number if available, and source.	Not Applicable	

Human research participants	Information included in the manuscript?	In which section is the information available? (Reagents and Tools Table, Materials and Methods, Figures, Data Availability Section)
If collected and within the bounds of privacy constraints report on age, sex and gender or ethnicity for all study participants.	Not Applicable	

Core facilities	Information included in the manuscript?	In which section is the information available? (Reagents and Tools Table, Materials and Methods, Figures, Data Availability Section)
If your work benefited from core facilities, was their service mentioned in the acknowledgments section?	Yes	Acknowledgements and Materials and Methods

Design

Study protocol	Information included in the manuscript?	In which section is the information available? (Reagents and Tools Table, Materials and Methods, Figures, Data Availability Section)
If study protocol has been **pre-registered, provide DOI in the manuscript**. For clinical trials, provide the trial registration number **OR** cite DOI.	Not Applicable	
Report the **clinical trial registration number** (at ClinicalTrials.gov or equivalent), where applicable.	Not Applicable	

Laboratory protocol	Information included in the manuscript?	In which section is the information available? (Reagents and Tools Table, Materials and Methods, Figures, Data Availability Section)
Provide DOI OR other citation details if **external detailed step-by-step protocols** are available.	Not Applicable	

Experimental study design and statistics	Information included in the manuscript?	In which section is the information available? (Reagents and Tools Table, Materials and Methods, Figures, Data Availability Section)
Include a statement about **sample size** estimate even if no statistical methods were used.	Yes	Figure legends
Were any steps taken to minimize the effects of subjective bias when allocating animals/samples to treatment (e.g. **randomization procedure**)? If yes, have they been described?	Not Applicable	
Include a statement about **blinding** even if no blinding was done.	Not Applicable	No blinding was performed
Describe **inclusion/exclusion criteria** if samples or animals were excluded from the analysis. Were the criteria pre-established?<br><br>If sample or data points were omitted from analysis, report if this was due to attrition or intentional exclusion and provide justification.	Not Applicable	
For every figure, are **statistical tests** justified as appropriate? Do the data meet the assumptions of the tests (e.g., normal distribution)? Describe any methods used to assess it. Is there an estimate of variation within each group of data? Is the variance similar between the groups that are being statistically compared?	Yes	Figure legends and Materials and Methods Section

Sample definition and in-laboratory replication	Information included in the manuscript?	In which section is the information available? (Reagents and Tools Table, Materials and Methods, Figures, Data Availability Section)
In the figure legends: state number of times the experiment was **replicated** in laboratory.	Yes	Figure legend
In the figure legends: define whether data describe **technical or biological replicates**.	Yes	Figure legend

Ethics

Ethics	Information included in the manuscript?	In which section is the information available? (Reagents and Tools Table, Materials and Methods, Figures, Data Availability Section)
Studies involving **human participants**: State details of **authority granting ethics approval** (IRB or equivalent committee(s), provide reference number for approval.	Not Applicable	
Studies involving **human participants**: Include a statement confirming that **informed consent** was obtained from all subjects and that the experiments conformed to the principles set out in the WMA Declaration of Helsinki and the Department of Health and Human Services Belmont Report.	Not Applicable	
Studies involving **human participants:** For publication of **patient photos**, include a statement confirming that consent to publish was obtained.	Not Applicable	
Studies involving experimental **animals**: State details of **authority granting ethics approva**l (IRB or equivalent committee(s), provide reference number for approval. Include a statement of compliance with ethical regulations.	Yes	Materials and Methods
Studies involving **specimen and field samples:** State if relevant **permits** obtained, provide details of authority approving study; if none were required, explain why.	Not Applicable	

Dual Use Research of Concern (DURC)	Information included in the manuscript?	In which section is the information available? (Reagents and Tools Table, Materials and Methods, Figures, Data Availability Section)
Could your study fall under dual use research restrictions? Please check biosecurity documents and list of **select agents and toxins** (CDC): https://www.selectagents.gov/sat/list.htm	Not Applicable	
If you used a select agent, is the security level of the lab appropriate and reported in the manuscript?	Not Applicable	
If a study is subject to dual use research of concern regulations, is the name of the **authority granting approval and reference number** for the regulatory approval provided in the manuscript?	Not Applicable	

Reporting

The MDAR framework recommends adoption of discipline-specific guidelines, established and endorsed through community initiatives. Journals have their own policy about requiring specific guidelines and recommendations to complement MDAR.

Adherence to community standards	Information included in the manuscript?	In which section is the information available? (Reagents and Tools Table, Materials and Methods, Figures, Data Availability Section)
State if relevant guidelines or checklists (e.g., **ICMJE, MIBBI, ARRIVE, PRISMA**) have been followed or provided.	Not Applicable	
For **tumor marker prognostic studies,** we recommend that you follow the **REMARK** reporting guidelines (see link list at top right). See author guidelines, under 'Reporting Guidelines'. Please confirm you have followed these guidelines.	Not Applicable	
For **phase II and III randomized controlled trials**, please refer to the **CONSORT** flow diagram (see link list at top right) and submit the CONSORT checklist (see link list at top right) with your submission. See author guidelines, under 'Reporting Guidelines'. Please confirm you have submitted this list.	Not Applicable	

Data Availability

Data availability	Information included in the manuscript?	In which section is the information available? (Reagents and Tools Table, Materials and Methods, Figures, Data Availability Section)
Have **primary datasets** been deposited according to the journal's guidelines (see 'Data Deposition' section) and the respective accession numbers provided in the Data Availability Section?	Not Applicable	
Were **human clinical and genomic datasets** deposited in a public access-controlled repository in accordance to ethical obligations to the patients and to the applicable consent agreement?	Not Applicable	
Are **computational models** that are central and integral to a study available without restrictions in a machine-readable form? Were the relevant accession numbers or links provided?	Not Applicable	
If publicly available data were reused, provide the respective **data citations in the reference list**.	Not Applicable	
